# Nanocarrier-mediated cancer therapy with cisplatin: *A meta-analysis with a promising new paradigm*

**DOI:** 10.1016/j.heliyon.2024.e28171

**Published:** 2024-03-27

**Authors:** Ranmali Ranasinghe, Michael Mathai, Mohammed Abdullah Alshawsh, Anthony Zulli

**Affiliations:** a Institute for Health and Sport, College of Health and Medicine, Victoria University, Melbourne, Victoria, Australia; b Department of Paediatrics, School of Clinical Sciences, Faculty of Medicine, Nursing and Health Sciences, Monash University, 246 Clayton Road, Clayton, VIC, 3168, Australia; c Department of Pharmacology, Faculty of Medicine, Universiti Malaya, Kuala Lumpur, 50603, Malaysia

**Keywords:** Cisplatin, Nanocarriers, Nanotechnology, Nanomedicine, Meta-analysis, Cancer cell viability, Tumour volume, IC50, Biodistribution of platinum in vital organs

## Abstract

**Aims:**

Cisplatin is a frontline chemotherapeutic utilized to attenuate multiple cancers in the clinic. Given its side-effects, a new cisplatin formulation which could prevent cytotoxicity, metabolic deficiencies and metastasis is much needed. This study investigates whether nanocarriers can provide a better mode of drug delivery in preclinical cancer models seeking a potent anticancer therapeutic agent.

**Materials and methods:**

The PubMed database was searched, and 242 research articles were screened from which 94 articles qualified for selection from those published by December 31, 2023 and the data was synthesized using the Review Manager software.

**Key findings:**

Cisplatin encapsulated as a nanomedicine confirmed the versatility of nanocarriers in significantly diminishing cancer cell viability, half maximal inhibitory concentration, tumour volume, biodistribution of platinum in tumours and kidney; at *p* < 0.00001 and a 95% confidence interval.

**Significance:**

An estimated 19.3 million global cancer incidence is reported with 50% mortality worldwide for which nanocarrier-mediated cisplatin therapy is most promising. Our findings offer new vistas for future cancer treatment when combined with chemo-immunotherapy that utilizes the recently advanced nanozymes.

## Graphical abstract

Created with BioRender.com.

**Figure:**
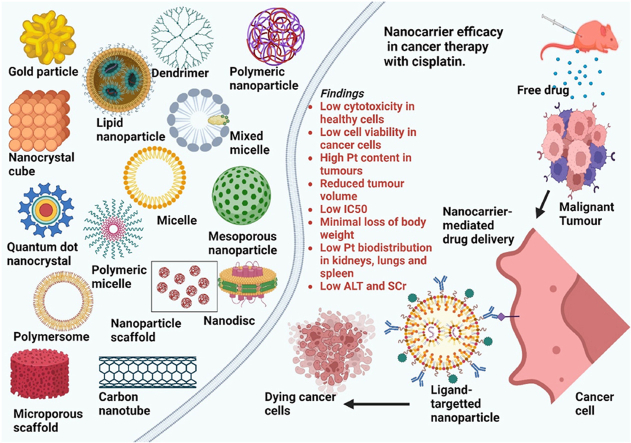

## 1 Introduction

### 1.1 Background

Cisplatin is a very effective chemotherapeutic agent that has the potential to attenuate tumour multiplication and metastasis in a spectrum of different cancers [1]. Despite its century-long history in cancer treatment, it is accompanied by many adverse effects that downgrade its therapeutic value and has made clinicians to cautiously prescribe it to the patients [1]. Cisplatin still does not possess the ability to fully suppress tumorigenesis, prevent excessive drug efflux, reduce severe toxicity in multiple organs, attenuate detrimental systemic pathophysiology, chemoresistance, and inhibit various cancer-related biochemical and immune pathways [2]. For this reason, cisplatin is utilized either as an adjuvant or in a combination of chemotherapeutics and/or radiation therapy, which can only extend the lifespan probably up to 5 years [3]. Cisplatin-treated major cancers comprise of respiratory, urinogenital, head and neck, skin, and enteric neoplasms [4], which receive single intravascular injections of two to three-weekly cycles at a high dose (50–100 mg/m^2^) [5] that is virtually intolerable with nausea, vomiting, diarrhoea, weight loss due to loss of appetite, accompanied by renal, hepatic, and nervous system failure [6]. Given this background of cisplatin as an anti-tumour medication, various drug delivery methods have been introduced, of which, nanocarrier-mediated cisplatin delivery is still the focus of experimental anticancer therapy [7].

A plethora of research studies have reported the advantages of nanotechnology-based cisplatin therapy as opposed to the conventional free drug treatment in both *in vitro* and *in vivo* preclinical models, which are promising alternatives that could be developed into clinical trials if a comprehensive evaluation is carried out [8]. The injected free drug travels around the body in blood plasma and undergoes cellular uptake, then rapid clearance and excretion through the renal system [9,10]. The conventional commonplace platinum-based therapeutic agents are prescribed up to 40–80% of the active ingredients; cisplatin, carboplatin and oxaliplatin, that are approved for cancer therapy by the FDA in the USA, but they significantly induce nephrotoxicity, neurotoxicity, and ototoxicity as major side effects [11]. These shortcomings have been overcome by utilizing nanoparticles for drug delivery by targeting the tumour sites, augmenting bioavailability, and minimising efflux [12]. They offer better strength, stability and biodistribution as drug carriers and produce low cytotoxicity in healthy cells [12]. The nanocarriers display preferential accumulation, enhanced retention, and controlled release at the tumours, making them superior deliverers compared to free intraperitoneal administration [13,14]. Cisplatin in nanocarriers are actively localized and internalized either by specific membrane-bound receptors or by endocytosis [15].

The free cisplatin treatment has not been successful 100 percent due to its untargeted delivery to malignant tumours, it's inability to specifically select tumour cells as well as chemoresistance of the tumour cells and those of the supplying vasculature [16]. The platinum formulation itself develops serious side-effects, not allowing the free cisplatin therapy to achieve a state of complete remission [17]. One of the main disadvantages of free drug therapy is its passive targeting of the tumours [18]. Tumours are a complex microenvironment carrying an abundance of fibroblasts, collagen fibres, macrophages, and an impaired lymphatic drainage system from which the nanocarriers cannot be easily eliminated [19]. Hence their retention time within the tumours is prolonged [19]. The gap size of the endothelial cells and the size of cross-endothelial channels determine the exudation strength of the nanocarriers [20]. Thus most of the nanocarrier types that have been developed so far, are small (between 10 and 150 nm) with the ability to tether ligands on to their external surface which in turn can bind with the overexpressed receptors (folate, transferrin, endothelial growth factor receptors -EGFR, and integrins) on cancer cells [21]. As such, the structure and composition of the nanocarriers play a key role in their commercial reproducibility as novel drug deliverers in cancer treatment [22]. The EPR effect is further augmented by the nanocarrier size, where a smaller structure could move easily through the endothelium and are also easily removed by the kidneys although a larger size would not be long-lasting as they are phagocytosed by the host's immune cells [23]. The most useful of the wide and varied nanocarrier types include polymeric micelles, liposomes, dendrimers and inorganic -based nano formulations [24]. The polymeric micelles consist of a hydrophobic core and an external ring of hydrophilic polymeric material which allows for carrying a mix of drugs that enable active targeting, biocompatibility, biodegradation, increased solubility, stability, longer half-life, easy recognition of tumour cells in several cancer types (breast, prostate, lung, and gliomas) [25,26]. In polymeric micelles, the core forms by the self-assembly of amphiphilic copolymers in aqueous solution while the hydrophilic outer layer keeps it hydrated [27]. Most of the polymeric material that is commonly used in nanocarriers include polyethylene glycol (PEG), polylactic acid (PLGA) and chitosan [28].

The most commonly used nanocarrier is the liposome which was first developed in the early sixties decade [29]. It is endowed with features that have made it the most-researched of the nanocarriers because they are formed from natural phospholipids and cholesterol [30]. It has a hydrophilic inner core for loading hydrophilic drugs while the hydrophobic moieties allow hydrophobic drugs to be embedded in the double layers of the lipid membrane [31]. They are flexible structures that can be modified to suit the desired composition required for targeted drug delivery to tumours [32] The liposomes can carry anti-cancer drugs within its core or the outer coat, which allows for easy integration into tumour tissues owing to its natural lipid composition [33]. The liposome was the first nanocarrier that was approved for anti-cancer drug delivery by the US food and drug agency (FDA) in 1995, coated with Doxorubicin, hence named Doxil which had high biocompatibility, longer half-life, and no immunogenicity [34]. Dendrimer nanocarriers are a novel drug delivery mode because they carry multiple functional groups on the surface and are composed of highly branched macromolecules [35]. Their multifunctionality has made them highly useful in targeting a multitude of receptors simultaneously, thus accessing several different types of malignant cells [36]. They have high solubility, low polydispersity, and adaptable cell surface chemistry [37]. The inorganic nanocarriers comprise the gold, mesoporous silica, carbon nanotubes, quantum dots, and magnetic nanocarriers to name a few which are a group of versatile drug delivery systems that are utilized as anti-cancer treatment [38]. These possess therapeutic value given their stability, small but adjustable size, shape, specific surface area, modifiable surface structures, and magnetism, all of which contribute to their comprehensive strength in cancer therapy [22].

Compared to conventional chemoradiation therapy which has many adverse effects (such as hair loss, suppression of bone marrow and derivatives and gastrointestinal complications), nanomedicine has achieved remarkable progress as an anti-neoplastic treatment in the recent decades [39]. It has shown promise in enhancing chemotherapy through drug synergism, gene therapy and immunotherapy [40]. The first phase 1 clinical trial with nanoparticles containing small interfering RNA (siRNA) as a treatment strategy for solid tumours got underway in 2010 which was followed by several more clinical studies [41]. The introduction of hybrid nanoparticles offer a multifunctional nano platform [42] which combines the favourable properties of several nanoparticle types for overcoming multidrug resistance, minimising off-target cytotoxicity and the inhibition of the membrane-bound efflux transporters on breast, ovarian and prostate cancer cells [43,44]. Apart from the EPR effect, the targeting of the tumours with nanocarriers may inhibit energy metabolism such as glycolysis which is required to maintain the higher tumour cell proliferation rate [45]. Glycolysis favours an acidic microenvironment that reduces the tumour pH level thereby inducing the low-pH -sensitive nanoparticles to sustainably release its drug content [45].

The particle size is an integral aspect of each nanocarrier type and its fate once inside the body [70]. There are many different ways in which the particle size would contribute to its intended biological functions. The nanocarrier size should be small enough to escape being destroyed by the reticuloendothelial system and should not be larger because it then faces phagocytosis or passing into the tissue fluid through the gaps in the capillary endothelium and be eliminated by the kidneys [71]. Other prime factors are that the size influences the encapsulating efficiency, drug loading capacity, and cellular uptake of the nanocarriers and its drug releasing kinetics [72]. The suitable size of a nanocarrier is between 100 and 150 nm [70], which is close to the average mean diameter (159 nm) of the different nanocarrier types included in Table 1. Similar to the size, the shape and the texture of the surface also determine the efficiency of the nanoparticles for drug delivery [73]. The most suitable shape is a sphere which has the largest relative surface area and having a decorated or rough surface increases the nanocarrier efficiency by tethering many ligands that bind with most of the overexpressed receptor types present on cancer cells [74]. Simple regression analysis of the data gathered in this study reveals a direct relationship between the nanocarrier size and tumour inhibition percentage (Fig. 2a) [75] as well as the drug releasing efficiency as a percentage (Fig. 2c) significant at p < 0.0001 [76]. Additionally, there is a significant positive linear relationship between TIR % and the DRR % (Fig. 2e) with an increasing releasing capacity that positively correlates with the tumour suppression rate [77].

The polydispersity index (PDI) reveals the heterogeneity of a given sample in terms of its size distribution. It is the ratio of the average molecular weight to its sample size (Mwt/n) in polymeric nanoparticles [70]. A PDI of less than 1 or below 0.2 is deemed favourable whereas monodisperses is indicated by a value of 1 or greater than 1 [70]. The average PDI of the given nanocarrier types in Table 1 is 0.167, which is an acceptable value [70]. The PDI is based upon the method and the materials used in the construction of nanocarriers such as the surfactant type and concentration, lipids used and the variability of the instrumental readings [78]. The overall electrical charge or the zeta-potential (ZP) is a crucial physical property because the biocompatibility and the cellular uptake rests on the uniform dispersion of the nanoparticles in a given suspension [78,79]. A higher charge indicates higher stability and prevents particle aggregation [80]. The average electrical charge distribution of the samples in Table 1 is −11.5 mV (mV). The preferred zeta potential values above +30 mV indicates good stability [81]. It is the electrical charge at the slipping plane or the surface of the liquid phase that separates the nanocarrier material in a suspension [82]. An overall negative charge reveals the electrostatic attraction between cationic nanoparticles that have higher adsorption capacity [83].

The drug encapsulation efficiency percentage (EE%) directly reflects the concentration of the active ingredients of the drugs entrapped within the nanocarriers [84]. It is measured by standard techniques such as ultraviolet (UV) spectrophotometry and high-pressure liquid chromatography (HPLC) [85]. The concentration of the drugs present in the supernatant is an indirect method of calculating EE% and it is an important parameter when synthesizing nanocarriers [86]. It is affected by the drug solubility and prevents metabolic and chemical degradation of the drug-loaded nanoparticles [87]. Also it increases the permeability of the membranes surrounding the nanoparticles and reduces the non-specific binding of plasma proteins and aggregations of colloidal particles on to the drugs [88]. The EE% is also based upon the particle size, concentration and solubility of the polymeric material and the rate of solubilization of the polymer in the solvent [89]. The average EE% of the nanocarriers included in Table 1 is 73.605 %, indicating high drug entrapment efficiency [90].

**Fig. 1:**
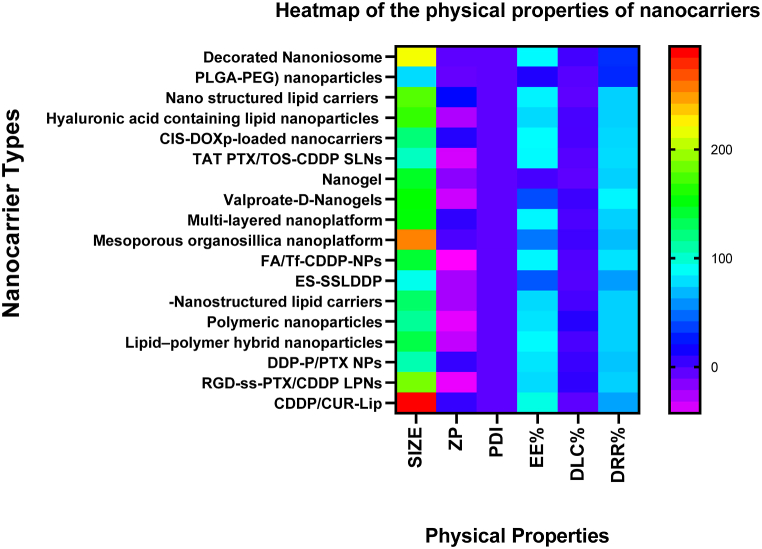
A heatmap of the physical properties [Size, Zeta potential (ZP) and Polydispersity index (PDI), Encapsulation efficiency percentage (EE%), Drug loading capacity percentage (DLC%), Drug releasing efficiency rate percentage (DRR%) of the nanocarrier types in Table 1. Refer Table 1 for the abbreviations.

The drug loading capacity of the nanocarriers is the amount of drug entrapped per unit weight of the nanocarrier [91]. It indicates the increase in mass of the nanocarrier on account of the drug that is imbibed into the nanoparticles [92]. Some common mechanisms of drug loading include adsorption, electrostatic forces, and/or hydrophobic interactions [93]. The drug releasing rate expressed as a percentage (DRR%) varies with different nanocarrier types [94]. It occurs mainly with the erosion of the nanocarrier matrix and is commonly measured by the dialysis method [95]. The DRR% is influenced by the pH of the medium where certain nanocarrier types require an acidic pH to function efficiently [96]. The entrapped drug is released initially as a burst which eventually becomes a sustainable, smooth flow [97]. The encapsulation efficiency, the drug loading capacity, and the drug releasing rate, all of which that are evaluated as percentages per the mass of the nanocarrier are important physical parameters that underpins the overall efficiency of a given nanocarrier as a drug delivery modality, to the tumours [98]. The average EE%, DLC%, DRR% and TIR% are 73.605%, 5.58%, 78.795% and 67.85% respectively, for the 18 nanocarrier types included in Table 1. Although these physical properties of the nanocarriers for optimising targeted drug delivery to cancers are advantageous, there is the disadvantage of nanoparticles crossing the blood-brain barrier and eventually releasing compounds that are toxic to the brain [99] The heatmap (Fig. 1) summarizes the six most relevant physical properties of the nanocarriers that included 18 different nanocarrier types that were experimented in xenografted mouse models of cancer.

**Fig. 2:**
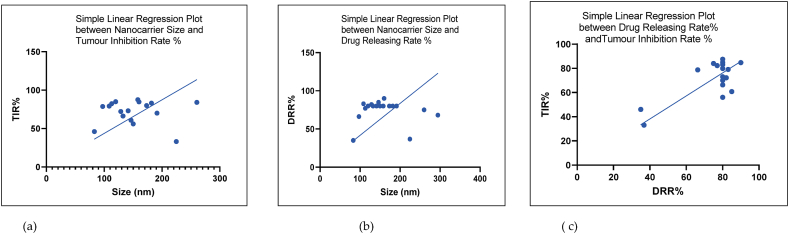
(a–c): Simple linear regression plots of nanocarrier size with tumour inhibition rate percentage (TIR%), drug releasing efficiency rate percentage (DRR%), DRR% with TIR% (a), (c) and (e) respectively.

A Meta-analysis is a statistical analysis tool that synthesizes quantitative evidence on a given research question by combining relevant results from multiple, independent studies which produces an effect size with a large power [100]. A large number of studies can be used to obtain and analyse the qualitative outcomes after following data selection and synthesis, assessing the risk of bias, and calculating the standardized mean difference (SMD) statistic to plot both forest and funnel plots which can elucidate the effect of a certain factor pertaining to a research question [101]. A Meta-analysis is a method of retrospective screening of data that comprises of an evaluation of pooled data which must be done in the context of a post-systematic review that follows a rigorous screening process [102]. One effect size is thus determined using statistical software which is specifically designed and relies on a specific protocol that defines all the relevant inclusion and exclusion criteria, to produce the most pertaining eligibility criteria [103,104]. This method is useful when there are either conflicting results on the same study topic or when a definite conclusion is not present. This is because primary research studies are considered unreliable due to their small sample size, which limits their statistical power [105]. Among the benefits of conducting a meta-analysis are increased reliance due to increased statistical power not had in an individual study and increased precision of the estimate at defined confidence intervals [106]. It also reduces various risks of bias and allows those risks to be corrected whilst investigating reasons for the differences in risk estimates and elucidating patterns of risks among the studies [106]. Therefore, a meta-analysis is deemed an overall accurate estimate of an effect size that pertains to a research question, obtained from pooling data and collectively analysing a very large number of studies [107].

We have selected seven distinct factors that are relevant to our research question, which is to determine the superiority and effectiveness of nanocarrier-mediated cisplatin delivery in comparison to free cisplatin administration in preclinical cisplatin-treated cancer models published in the last decade. The impact of nanocarrier-tethered cisplatin and free drug delivery on (i) cell viability [46] (ii) half-maximal inhibitory concentration (IC50) [46], (iii) tumour volume [108], (iv) biodistribution of platinum (Pt) in the tumours [109], and (v) vital organs [55], (vi) haematological parameters of nanocarrier biosafety [110] and (vii) the gain or loss in body weight of the mice [111] were studied. The cell viability percentage is an indication of the cytoprotective ability of the nanocarrier-mediated cisplatin delivery which is also an indirect estimate of survivability [112], or the proliferation ability of cancer cells and an approximate rate of apoptosis or cell death measured by MTT assay [113]. The half-maximal inhibitory concentration denoted by IC50, represents the concentration at which a given drug is potent in inhibiting a specific biological or biochemical function [114]. The lower the IC50, greater is the potency of the drug, which is then considered favourable, that signifies with how little of the substance, it is able to inhibit the biological function under investigation [114]. Similarly, the tumour volume is a commonly measured parameter in mouse models of cancer that evaluates cisplatin treatment, as the tumours shrink with increased cytotoxicity exerted by anticancer therapies [52]. The tumour volume increases in the control group when given a placebo or a free drug formulation whereas the nanocarriers possess the ability to augment the accumulation of chemotherapeutics owing to its enhanced pharmacokinetic properties [52]. The nanocarriers have confirmed their superior ability for tumour targeted delivery of therapeutics with long-term stability, low cytotoxicity, sustained release, longer retention, biodegradability, and less side effects that enhance the antitumour efficacy of a given drug [115,116]. The biodistribution of platinum in the vital organs is a measure of the amount of drug that enters the systemic circulation which diffuses into the vital organs (heart, liver, and kidneys) thus exposing the otherwise healthy organs to high chemotherapy-related toxicity [117]. The nanomedicine approach has been effective in shielding the vital organs against unwarranted exposure to strong anticancer therapeutics and assist in maintaining overall homeostasis in the cancer-afflicted individuals. The major drawback of cisplatin therapy is the rapid loss of body weight that is identified as its most detrimental side effect [118]. Cisplatin treatment induces loss of appetite and general malaise in addition to nausea, vomiting, and extreme sickness which rapidly deteriorate the patient's general wellbeing [119]. Nanocarrier-mediated drug treatment was found to be highly effective in preclinical models in containing the loss of weight by either maintaining the body weight at the same level before the commencement of therapy or by producing even a slight increase in the subject's weight [120].

We report for the first time a meta-analysis with 7 primary outcomes which reflect nanocarrier efficiency as opposed to free drug administration in *in vivo* preclinical cancer models of cisplatin. The association of cell viability, IC50, tumour volume, biodistribution of platinum (Pt) in tumours and vital organs and the body weight of the mice have produced a clear mandate for nanocarrier use. As such, nanocarriers would promote health benefits to resist the detrimental effects of free cisplatin, if utilized in formulating better drug delivery applications from nanotechnology combined with favourable pharmacokinetics of cisplatin.

### 1.2 Description of the condition

The primary mechanism of cisplatin chemotherapy is to disrupt DNA synthesis in tumour cells thereby preventing cell proliferation of the cancers [121]. Cisplatin binds to nuclear deoxyribonucleic acid (DNA), at the N7 reactive centre of purine residues by forming 1,2 intra-strand crosslinks causing DNA damage [122]. It interferes with the G0/G1 phase of the cancer cell cycle, thereby inducing apoptotic cell death [123]. The plasma platinum content exerts a direct effect on the cisplatin chemotherapeutic mechanism in attenuating tumour proliferation and subsequent metastases [124]. The studies which used the Inductively Coupled Plasma (ICP) method to quantify the plasma platinum concentration were only selected in order to reduce detection and reporting bias in the selected studies.

### 1.3 Description of the intervention

Nanocarriers were first introduced in 1980 and were patented with a chemotherapeutic formulation in 1995 [125,126]. There is a plethora of research studies that have been published owing to its superiority over free drug administration in the field of nanomedicine. Possibly the term ‘nano’ was derived from its critical feature of having a submicron particle size such as being smaller than 500 nm [127]. A nanocarrier consists of a minute bilayer structure composed of a small molecule in which a soluble drug is encapsulated [128]. Nanocarriers have been mostly experimented within targeted delivery of chemotherapeutics into cancerous tumours [129] although it is yet to be established as a treatment modality in common clinical therapeutic use for attenuating cancer malignancies. There are around 70 nanomedicines that are approved by the US food and drug administration (FDA) and the European Medicines Agency since the year 1995 [130].There are three categories of nanocarriers that are based upon the nanomaterial that is used for its construction, namely, lipid-based, inorganic, and polymeric nanocarriers [131]. They have become popularised due to the composite advantage of incorporating both hydrophilic and hydrophobic moieties, high stability, high carrier capacity, and feasibility in administration *via* the oral, intravenous, and inhalation routes [132,133]. The main applications of nanocarriers in the sphere of nanomedicine consist of drug delivery, imaging, sensing, tissue engineering, in medical devices and sepsis treatment [134]. Nanocarriers are made in a variety of shapes and sizes named as, nanoparticles, niosomes, nanoliposomes, exosomes, nanogels, nano sponges, nanorods, micelles and dendrimers [135]. They possess a competitive advantage over free cisplatin therapy by displaying superior biocompatibility, biodegradability, non-immunogenicity, and high stability within cancerous tumours [136]. They are able to overcome the short blood circulatory time and fast removal by the reticuloendothelial system (RES) [137]. Moreover, surface decoration by bioactive material such as folic acid bestows the ability to resist quick elimination by the RES [138].

The nanocarriers have been a popular solution to minimize quick drug efflux from tumour tissue, acute systemic toxicity, expeditious clearance from the systemic circulation and chemo-resistant adaptability in cancers receiving cisplatin therapy. Cisplatin is a frontline chemotherapeutic utilized to attenuate multiple cancers in the clinic but is accompanied by severe, intolerable side-effects. The need to establish a safe and efficient drug delivery mode for cisplatin is imperative because an overall 19.3 million cancer incidence is reported with 10 million deaths worldwide [139] which include numerous cisplatin-treatable cancers.

### 1.4 Objectives

• To ascertain whether the nanocarrier-mediated cisplatin delivery has superior efficacy by overcoming the daunting obstacles encountered and surmounting the drug's ill-effects than the conventional free cisplatin treatment.
• To verify whether cisplatin treatment when delivered nanocarrier-bound is more effective at retention and assimilation within the tumours.
• To assess if it is comparatively more beneficial to the model in maintaining its overall general health.

## 2 Methods

### 2.1 Eligibility criteria for considering studies for this review

Only pre-clinical experimental studies which are case-controlled baseline studies on mouse spp., that have free cisplatin as the control with a duration no longer than 3–4 weeks and having no more than 3–4 treatment cycles were included in this meta-analysis. Mouse (*Mus muscularis*) aged 4–16 weeks and of both genders, weighing about 20–24 g (g), induced with cisplatin-treatable cancers, that were injected either subcutaneously or IP with cancer cell lines to produce xenograft murine models were utilized with a sample size that is equal to or more than 3 per treatment group. Nanocarriers containing cisplatin, belonging to nanoparticles, nanoliposomes, nanoniosomes, nanotubes, nanocubes, nanogels, and nanorods of size between 30 and 200 nm (nm) having positive or neutral zeta potential and poly dispersity index (PDI) between 0.1 and 1.0, that are internalized by endocytosis and scanned images obtained from transmission electron microscopy (TEM), and having a lower half-maximal inhibitory concentration (IC50) than the free drug were included.

### 2.2 Types of outcome measures

There are 7 primary outcomes that were evaluated with no secondary outcomes. They comprised of (i) low cell viability % in cancer cells/tumour tissue, (ii) lower IC50 value than free drug (at the concentrations of μg/ml or μM) (iii) reduced tumour volume (mm^3^), (iv) higher platinum (Pt) concentration in tumour cells (at μg/ml or μM/l), (v) lower Pt biodistribution in vital organs (heart, kidney, and liver (μg/g) and (vi) moderate body weight loss or weight gain (g).

### 2.3 Search methods for identification of studies

**Fig. 3:**
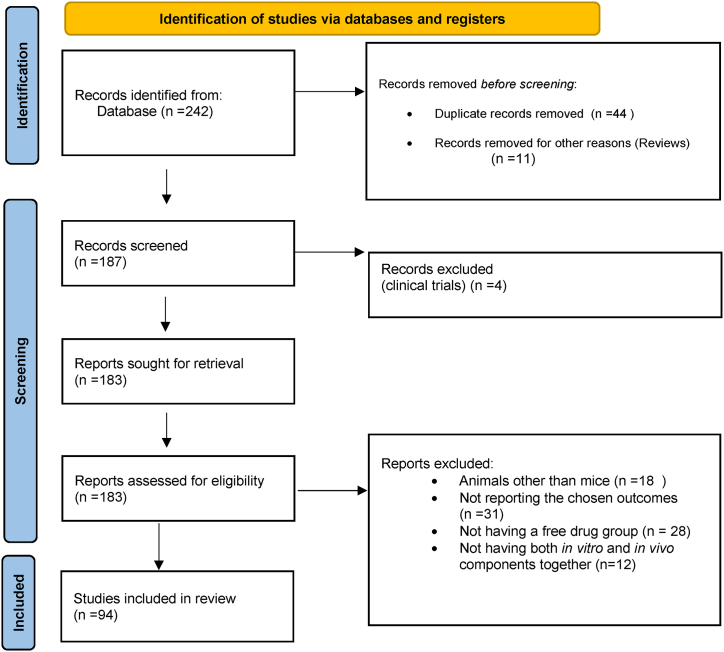
A modified version of the PRISMA statement of year 2020 used for obtaining the included studies [140].

The PRISMA and the Cochrane collaboration guidelines applicable to meta-analyses of interventional studies (Fig. 3) in preclinical mouse models and *in vitro* cell lines were adhered to in extracting data for this study. The PubMed was searched on October 22, 2022 using MeSH and Boolean terms (nanocarrier OR nanotechnology AND mouse OR animal studies AND cisplatin) for electronic databases. The first author conducted the search, identification, and screening of the manuscripts while another author independently repeated the screening and selecting the studies as per the eligibility criteria. No country restrictions were introduced. All bibliography were manually screened to identify any articles that may have been omitted.

#### 2.3.1 Electronic search history

The full search strategy is based on the search components using search filters in the PubMed database. Example: MeSH terms on PubMed: Animals, cell line, tumour, cisplatin, liposomes, therapeutic use, mice, neoplasms, drug therapy and the following search terms were used: (“Nanocarriers” OR “Nanoliposomes” OR “Nanoniosomes”) AND (‘Cisplatin-treated cancers” OR “cisplatin for neoplasms” OR “cisplatin for cancer therapy”). In addition, Boolean terms for electronic databases ((nanocarrier OR nanotechnology) AND (mouse OR animal studies) AND cisplatin) were also used.

### 2.4 Data collection and analysis

The first author read the full text articles identified in the database searches and extracted the data, as per the criteria defined. The characteristics of the included studies are defined in Table 1. Reviews, conference papers, posters, mini reviews, short communications, and letters were excluded. Data analysis was carried out by creating a forest plot, parametric paired student t-test, estimation plots and multiple regression analysis for each of the primary outcomes that were evaluated.

### 2.5 Data extraction and management

A standardized, pre-piloted form (data extraction spreadsheets) was used to extract data from the included studies for assessment of study quality and evidence synthesis. The extracted information included baseline characteristics, details of the intervention and control conditions, study methodology; outcomes and times of measurement; suggested mechanisms of intervention action; and information for an assessment of the risk of bias. The data extraction was carried out according to pre-specified inclusion criteria from only the peer-reviewed primary research studies that were only in the English language.

### 2.6 Assessment of risk of bias in included studies

All included studies located were screened for a quality of bias assessment by two of the authors according to the criteria suggested in the Cochrane guidelines as outlined in the Cochrane Handbook for Systematic Reviews of Interventions. Each included article was reviewed, and the risk of bias assessment procedure was conducted and recorded using the SYRCLE's tool. For the articles that were indicated as having high-risk of bias, a sensitivity analysis was performed and the results of estimate in the presence and absence of these studies were reported.

#### 2.6.1 Assessment of reporting biases

Assessing publication bias involves examining whether there is a systematic difference between published studies and unpublished studies that have the same research question. One commonly used method for assessing publication bias is by using funnel plots, which visually display the relationship between effect size estimates and study precision. To interpret this plot: studies with larger sample sizes will have smaller standard errors and will be plotted near the top of the graph, while studies with smaller sample sizes will have larger standard errors and will be plotted near the bottom of the graph. If there is no publication bias, the studies will be evenly distributed around the overall effect size estimate, forming a symmetrical funnel shape, while if there is publication bias, studies with larger effect sizes or smaller standard errors may be more likely to be published, resulting in an asymmetrical funnel plot.

### 2.7 Data synthesis and measures of treatment effect size

The results were synthesized using both a narrative approach and quantitative meta-analysis which was presented in the results and interpreted in the discussion section as well. For the quantitative meta-analysis, RevMan 5.4 version software was utilized to conduct the analysis. Since all the evaluated primary outcomes consisted of continuous data, a Standardized Mean Difference (SMD) was calculated. As heterogeneity was expected among the effect sizes in the included studies, a random-effects model was employed, and a forest plot was produced to display the results. The random-effects model is often preferred over the fixed-effects model in the presence of heterogeneity, as it considers the variability in effect sizes across studies, providing a more cautious estimate of the true effect size. Different units of analysis were included if the minimum individual participant number exceeded 10. Missing data was requested from the study authors up to two times. If there was no response from the authors, the study was excluded from the analysis. Two authors extracted the data independently, and any discrepancy identified was resolved through discussion with the third author where necessary. The meta-analysis was registered at the PROSPERO register in January 2023 (Available from: https://www.crd.york.ac.uk/prospero/CRD42023369333).

## 3 Results

### 3.1 *The included studies comprise of* 94 *research articles*

**Fig. 4:**
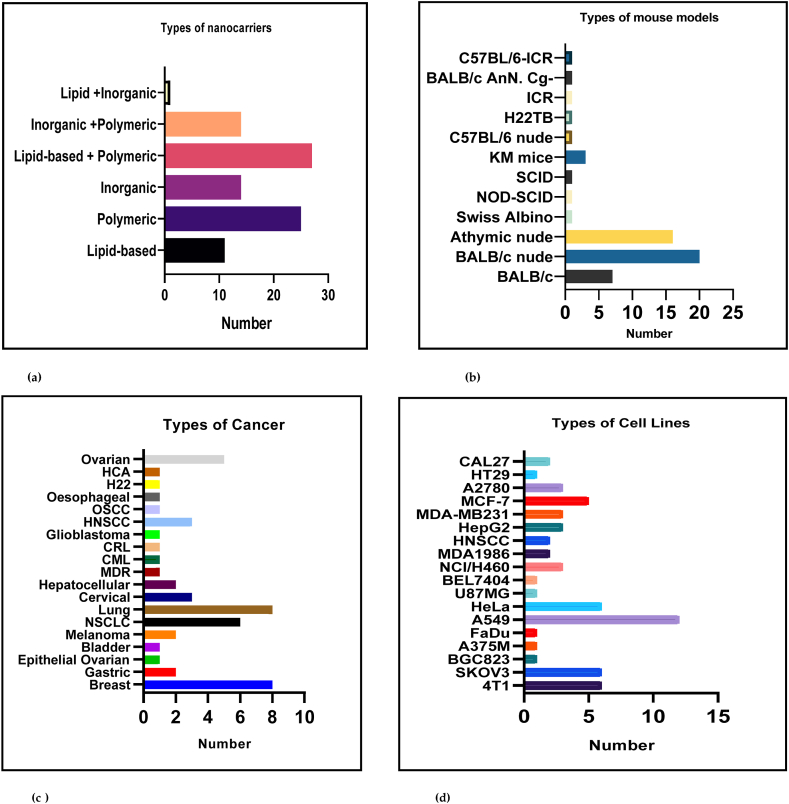
**(a**–**d): The different types of (a) nanocarriers, (b) mouse models, (c) cancers and (d) cancerous cell lines that are included in this meta-analysis. Legend:** C57BL/6 is a C57 black 6, C57 black 6/ICR -C57 black 6 inbred Swiss albino, BALB/c is an albino, laboratory-bred strain of the house mouse, H22-bearing hepatocellular carcinoma cell line, BALB/c nude -laboratory mouse from a strain with a genetic mutation that causes a deteriorated or absent thymus (athymic or immunodeficient) KM- The Chinese Kunming (KM) mouse colony, the largest outbred Chinese mouse stock, SCID -severe combined immunodeficiency disease-bearing mice, NOD-SCID- Non-obese diabetic/severe combined immunodeficiency-a model system to study the engraftment and mobilization of human peripheral blood stem cells, Swiss Albino- Swiss inbred strain used widely in research as a general purpose strain, Athymic nude- A type of laboratory mouse that is hairless, lacks a normal thymus gland, and has a defective immune system because of a genetic mutation often used in cancer research because they do not reject tumour cells, from mice or other species, HCA -hepatocellular carcinoma cell line, H22- mouse hepatocellular carcinoma cell line which is a tumour of the liver, OSCC- Oral squamous cell carcinoma (OSCC) is the most common oral cancer worldwide, HNSCC- Head and neck squamous cell carcinomas (HNSCCs) develop from the mucosal epithelium in the oral cavity, pharynx and larynx and are the most common malignancies that arise in the head and neck, CRL-colorectal cell line derived from tumours at various levels of differentiation and stages of development, CML-Chronic myeloid leukemia (CML) is a myeloproliferative disorder of hematopoietic stem cell origin, MDR-multidrug resistance which is acquired resistance to one anti-tumour drug usually showing resistance to other anti-tumour drugs, NSCLC-non-small-cell lung cancer (NSCLC) including squamous cell carcinoma and adenocarcinoma that accounts for the majority (85%) of lung cancer diagnoses, CAL 27- an oral adeno-squamous carcinoma cell line, HT22-human colon cancer cell line, A2780-an ovarian cancer cell line, MCF-7- Michigan Cancer Foundation (MCF) and is the most studied human breast cancer cell line in the world, MDA-MB 231- an epithelial, human breast cancer cell line, HepG2-a human liver cancer cell line, MDA-1986-Muscular Dystrophy Association (MDA) gene -derived cell line that causes Duchenne muscular dystrophy when mutated, NCI/H460-hypotriploid human cell line derived from pleural fluid of large cell lung carcinoma, BEL7404-a hepatoma cell line, U87MG-a cell line with epithelial morphology that was isolated from malignant gliomas, HeLa- the first immortal human cell line derived from a cervical cancer, A549-most commonly used human non-small cell lung cancer cell line for both basic research and drug discovery, FaDu-a cell line with epithelial morphology derived from a hypopharyngeal tumour. A375M − a cell line exhibiting epithelial morphology isolated from malignant melanoma, BGC 823-originally thought to be human gastric adenocarcinoma but found cross contaminated with cervical cancer cells, SKOV 3- a human ovarian cancer cell line with epithelial-like morphology, 4T1-a murine mammary carcinoma cell line from a BALB/cfC3H mouse that closely mimics stage IV human breast cancer.

A total of 242 articles were retrieved from the PubMed database out of which 44 were identified as duplicates, 4 were clinical trials, and 11 were reviews. Upon full-text examination, additional 146 studies were excluded for various reasons including, absence of a free drug control (n = 28), utilization of animals other than mice (n = 18), being single *in vitro* or *in vivo* studies (n = 13), reporting incompatible and irrelevant research data, no sample size reported, articles published before the year 2012, and absence of cisplatin and not being cancer studies (30). The final number of studies included after screening was 94 and their basic characteristics are described in table S1. The article retrieval was done by electronic searches with applying the Boolean search method and MeSH keywords. These studies compared the free cisplatin treatment for attenuating cancer in preclinical cancer models with nanocarrier-mediated cisplatin delivery. The aim was to assess the efficiency of the nanocarriers in cisplatin therapy and the results displayed an overall effectiveness in all the primary outcomes that were tested. This study included six different types of nanocarriers which were classified as lipid-based, polymeric, inorganic, lipid-based and polymeric, the inorganic and polymeric and lipid-based and inorganic (Fig. 4a). This analysis was carried out using 12 different xenograft mouse models (Fig. 4b), in which 19 different cancerous tumour types were induced (Fig. 4c), and also utilized 18 *in vitro* cancer cell lines (Fig. 4d) on which cellular cytotoxicity studies had been performed. The average dose of free cisplatin that was injected into mice through the intraperitoneal or subcutaneous route varied between 4 and 10 mg/ml with an average study duration of 14 days.

### 3.2 A total of seven primary outcomes are reported

A total of seven primary outcomes were tested and the overall results in each outcome highlighted the superiority of the nanocarriers over free cisplatin delivery in all the 94 murine models that were examined.

#### 3.2.1 Nanocarrier-mediated cisplatin delivery significantly suppressed cancer cell viability in vitro

**Fig. 5:**
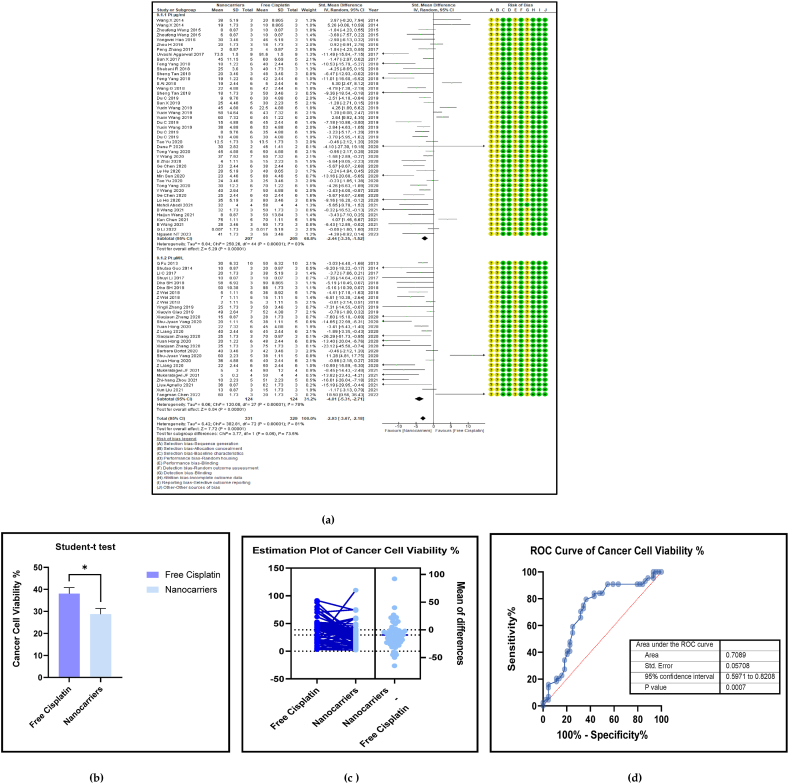
(a): Forest plot of cell viability percentage, (b) Paired *t*-test as a bar graph (c) an estimation plot and (d) Receiver Operator Characteristics (ROC) Curve of cancer cell viability % in nanocarrier-mediated cisplatin delivery compared with free cisplatin treatment.

A distinct feature of nanocarrier-mediated anticancer therapy is the high suppression of cancer cell proliferation. The data extracted from the individual studies were obtained from performing *in vitro* cancer cell proliferation assays using cancer cell lines, and either presented as a bar graph or a line graph. Fig. 5 (a, b, c, and d**)** show the forest plot, the paired *t*-test, the estimation plot, and the Receiver Operator Characteristic curve (ROC) for cell viability % of cancer cell lines. The use of nanocarrier-mediated cisplatin led to a significantly lower cell viability percentage in cancer cells compared to free cisplatin treatment. The overall pooled effect size was −2.93 (SMD) which had a 95% confidence interval (CI) of −3.67 to −2.18 (*p* < 0.00001), indicating strong statistical significance evidenced by both the forest plot and the bar graph depicting the results of the paired *t*-test (*p* < 0.01) (Table 2). The cancer cell viability percentage was calculated separately in two subgroups based on the units of cisplatin concentration added as micrograms per millilitre (μg/ml) and micromoles per litre (μM/L) with which the cell lines were incubated. The forest plot data, the paired *t*-test and the estimation plot reconfirmed the overall results obtained and consequently favour the nanocarriers over free cisplatin treatment.

**Table 1:** A cross-sectional quantitative analysis of the characteristics of nanocarrier types from the included studies. Legend: PDI- polydispersity index, EE%-encapsulating efficiency percentage, DLC-drug loading capacity percentage, NS-not specified.

Type and class of Nanocarrier	Size of Drug-loaded Nanocarrier (nm)	PDI of Drug-loaded Nanocarrier	Zeta Potential of Drug-loaded Nanocarrier (mV)	EE %	DLC %	Drug releasing efficiency %	CI	Tumour Inhibition Rate (TIR)	Drug dose of Cisplatin	Schematic Structure and References	
Decorated Nanoniosome (LIPID-BASED)	**224.5 ± 11.7**	**0.175 ± 0.024**	**NS**	**91.24 ± 1.32**	**6.9**	**36.78**	**0.7**	**NS**	**10 mg**		[46], [47]
Co-encapsulated cisplatin (CP) and paclitaxel (PTX) in poly (lactic-*co*-glycolic acid)-poly (ethylene glycol) (PLGA-PEG) nanoparticles (NPs) (POLYMERIC)	**82.9 + 2.7**	**0.1 + 0.01**	**−1.83 + 0.17**	**16.8±** **1.3**	**1.88 ± 0.1**	**35**	**NS**	**33**	**3.9 mg/kg**		[48,49]
Nano structured lipid carriers (NLC) (LIPID BASED)	**181.6 ± 3**	**0.21 ± 0.04**	**+26.3 ± 2.4**	**89.1±** **2.1**	**NS**	**80**	**0.81**	**46**	**1 mg**		[50,51]
Hyaluronic acid containing lipid nanoparticles (LIPID BASED)	**173.2 ± 5.9**	**NS**	**−21.5 ± 3.2**	**82.5±** **3.9**	**5.9 ± 0.8**	**80**	**NS**	**83**	**5 mg**		[52]
Cisplatin and Doxorubicin prodrug loaded nanocarriers (CIS-DOXp-loaded nanocarriers) (LIPID BASED)	**128.6 ± 3.2**	**0.196 ± 0.021**	**15.7 ± 1.7**	**92.1 ± 2.1**	**5.6 ± 0.6**	**82**	**0.57**	**79.9**	**5**–**10 mg**		[53,54]
*Trans*-activating transcriptional activator (TAT)- modified solid lipid nanoparticles (TAT PTX/TOS-CDDP SLNs) (POLYMERIC)	**108.6 ± 3.1**	**0.19 ± 0.04**	**−31.2 ± 2.7**	**91.1 ± 3.2**	**2.3 ± 0.3**	**83**	**0.646**	**72.2**	**10 μg/kg**		[55]
Nanogel (POLYMERIC)	**150–175**	**0.05-0.19**	**−12**	**6.4**	**NS**	**80**	**NS**	**79.14**	**4 mg/kg**		[56]
Valproate-D-Nanogels (POLYMERIC)	**160.1**	**0.167**	**−28.7**	**44.85**	**8.97**	**90**	**NS**	**56**	**6 mg/kg**		[57]
Multi-layered nanoplatform	**158**	**0.11**	**12.3**	**90**	**5**	**80**	**0.8**	**84.7**	**2 mg/kg**		[58]
Mesoporous organosillica shell-coated cisplatin nanoplatform (INORGANIC)	**260**	**NS**	**4.8**	**56.4±** **3.1**	**8.6 ± 1.7**	**75**	**0.3-0.9**	**87.5**	**2 mg/kg**		[59,60]
Folic acid (FA) and transferrin (Tf) modified cisplatin (CDDP) loaded nanoparticles (FA/Tf-CDDP-NPs) (POLYMERIC)	**146.2 ± 5.1**	**0.19 ± 0.03**	**−42.4 ± 3.3**	**90.2 ± 3.4**	**3.7 ± 0.4**	**85**	**NS**	**84**	**1 mg/kg**		[61]
Estrone-targeted PEGylated Liposomal DDP (ES-SSLDDP) (LIPID BASED POLYMERIC)	**97.3 ± 0.71 0**	**0.206 ± 0.005**	**−19.3 ± 0.36**	**47.7 ± 0.92**	**3.01 ± 0.04**	**66.33**	**NS**	**60.78**	**6 mg/kg**		[62]
NLC -Nanostructured lipid carriers (LIPID BASED)	**132.4 ± 5.3**	**0.158 ± 0.015**	**−19.3 ± 1.9**	**82.6 ± 3.9**	**6.6 ± 0.5**	**80**	**NS**	**78.7**	**10 mg/kg**		[63,64]
PNP- Polymeric nanoparticles (POLYMERIC)	**119.8 ± 4.4**	**0.163 ± 0.02**	**−35.6 ± 3.1**	**85.1 ± 3.6**	**15.1 ± 1.2**	**80**	**NS**	**66.3**	**10 mg/kg**		[63,65]
LPN- lipid–polymer hybrid nanoparticles (LIPID BASED POLYMERIC)	**141.2 ± 6.3**	**0.188 ± 0.027**	**−26.3 ± 2.6**	**91.3 ± 2.9**	**5.2 ± 0.6**	**80**	**NS**	**84.9**	**10 mg/kg**		[63,66]
DDP-P/PTX NPs -Cisplatin prodrug and paclitaxel co-loaded nanoparticles (INORGANIC)	**112.9 ± 3.5**	**0.15 ± 0.02**	**10.9 ± 1.3**	**85.3 ± 2.9**	**9.5 ± 0.7**	**77**	**0.562**	**73**	**5 mg/kg**		[67]
RGD-ss-PTX/CDDP LPNs- Redox sensitive lipid-polymer nanoparticles (LIPID BASED POLYMERIC)	191.3 ± 5.3	0.16 ± 0.03	**−37.2 ±** **3.9**	82.7 ± 4.1	12.3 ± 1.1	**80**	0.8	82.3	25 mg		[67,68]
CDDP and curcumin (CUR) co-loaded liposomes (CDDP/CUR-Lip) (LIPID-BASED)	**294.6 ± 14.8**	**0.119 ± 0.069**	**10.8 ± 2.5**	**99.5 ± 1.5**	**NS**	**68.2**	**0.9**	**70**	**1 mg/kg**		[69]
Average values of each column	**159**	**0.167**	**−11.4**	**73.6**	**5.58**	**78.795**		**67.85**

**Table 2:** Summary statistics of the cancer cell viability % between free cisplatin delivery compared with the nanocarriers.

OUTCOME	STUDIES	# Mice	SMD	95% CI	Z SCORE	*P* < *0.05*	TAU^2^	I^2^%	Chi SQ	DF	*P* < *0.05*	*STUDENT t-TEST p VALUE*
Subgroup 1 Pt μg/ml	29	412	−2.44	[-3.35, −1.52	5.20	*0.00001*	6.84	83	258.26	44	*0.00001*	
Subgroup 2 Pt μM/L	18	248	−4.01	[-5.31, −2.71]	6.06	*0.00001*	6.04	78	120.8	27	*0.00001*	
Overall Cell Viability %	47	660	−2.93	[-3.67, −2.18]	7.72	*0.00001*	6.42	81	382.81	72	*0.00001*	*0.0146*

#### 3.2.2 The half-maximal inhibitory concentration (IC50) of the cancer cells was significantly reduced by the nanocarriers

**Fig. 6:**
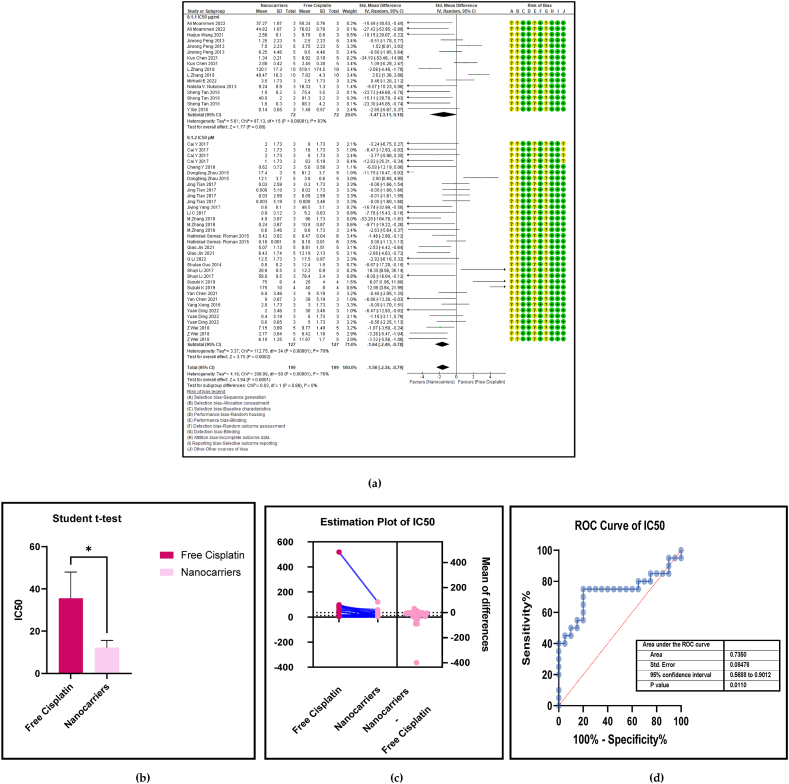
(a): Forest plot, (b) paired *t*-test as a bar graph (c) estimation plot and (d) ROC curve of IC50 of the cancer cells in nanocarrier-mediated cisplatin delivery compared with free cisplatin treatment.

IC50 is the half-way concentration of cisplatin that is required for the total inhibition of a given biological or a biochemical function. In this instance, it is the half-way concentration of platinum, required to completely kill the total volume of a given cancer cell line *in vitro*. Fig. 6 (a, b, c, and d) shows the forest plot, paired t-test-depicting bar graph, and estimation plot for the IC50 of cancer cell lines between the nanocarrier-mediated cisplatin delivery compared with free cisplatin treatment. The data have revealed (Table 3) that the use of nanocarrier-mediated cisplatin led to a significantly lower IC50 in cancer cells compared to free cisplatin treatment, with an overall pooled effect size of −1.56 (SMD) and a 95% confidence interval of −2.34 to −0.79 (*p* < 0.0002), indicating strong statistical significance. It substantiates that the effect sizes predominantly exhibit negative values, indicating a reduction compared to the control group. Consequently, this favours nanocarriers over free cisplatin treatment. Specifically, the effect sizes of the IC50 values in the nanocarriers mostly assume negative values compared to the control group, affirming that the nanocarrier IC50 values are lower than those in the free cisplatin treatment group.The cancer cell IC50 was calculated separately in two subgroups based on the units of cisplatin concentration as micrograms per millilitre (μg/ml) and micromoles per litre (μM/L) to which the cell lines were exposed. The paired *t*-test and the estimation plot reconfirmed the results obtained from the forest plot by displaying a significant difference (*p* < 0.01) between the nanocarriers and free cisplatin as shown in Fig. 6b and c. However there was a study that reported evaluating normal cells (fibroblasts) that were exposed to free Pt treatment against nanocarrier-bound Pt, the outcome of which had no morphological changes or viability issues even after exposing them to a high concentration of Pt (500 μg/mL) *in vitro* [141].

**Table 3:** Summary statistics for the cancer cell IC50 between free cisplatin delivery compared with the nanocarriers.

OUTCOME	STUDIES	# Mice	SMD	95% CI	Z SCORE	*P* < *0.05*	TAU^2^	I^2^%	Chi SQ	DF	*P* < *0.05*	*STUDENT t-TEST p VALUE*
Subgroup 1 Pt μg/ml	9	144	−1.47	[-3.11, 0.16]	1.77	*0.08*	5.61	83	87.13	15	*0.00001*	
Subgroup 2 Pt μM/L	17	254	−1.64	[-2.49, −0.78]	3.75	*0.0002*	3.37	70	112.75	34	*0.00001*	
Overall Cancer cell IC50	26	398	−1.56	[-2.34, −0.79]	3.94	*0.0001*	4.16	76	208.09	50	*0.00001*	*0.0233*

#### 3.2.3 The tumour volume was remarkably reduced with the nanocarrier-delivered cisplatin treatment

**Fig. 7:**
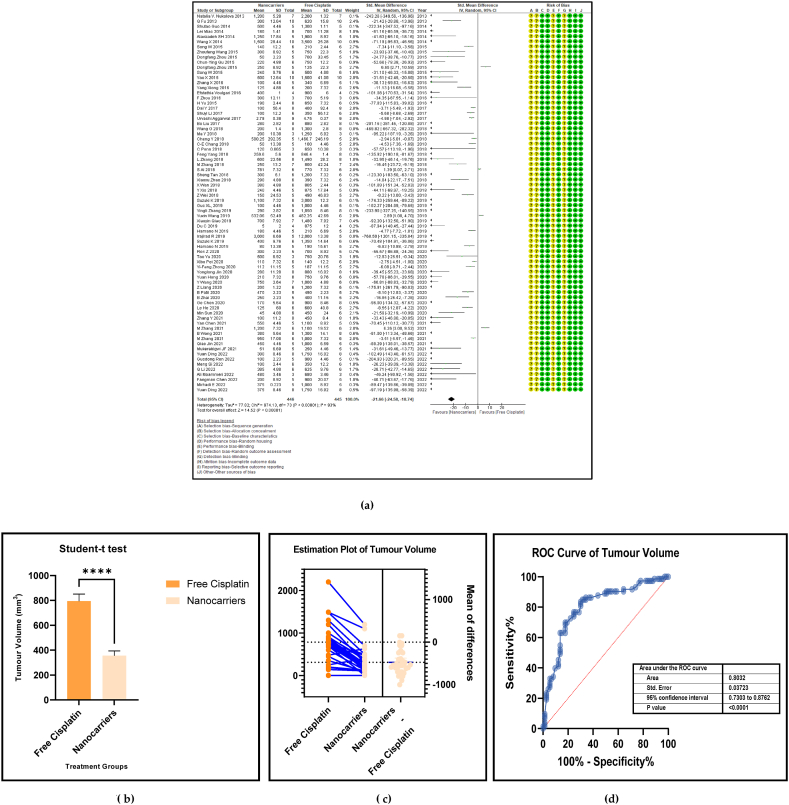
(a): Forest plot, (b) paired *t*-test as a bar graph (c) estimation plot and (d) ROC curve of the tumour volume in nanocarrier-mediated cisplatin delivery compared with free cisplatin treatment.

The shrinking of the tumour volume is a remarkable factor in anticancer treatment carried out with any suitable drug. A conspicuous reduction in the tumour volume indicates the potency of any anticancer drug in inhibiting tumour growth. Fig. 7 (a, b, c, and d**)** shows the forest plot, paired t-test-depicting bar graph, and the estimation plot for the tumour volume at sacrifice in the mouse models given nanocarrier-mediated cisplatin compared with free cisplatin treatment. The data revealed that the use of nanocarrier-mediated cisplatin led to a significantly lower tumour volume (mm^3^) in the mouse models compared to free cisplatin treatment. The overall pooled effect size was −19.38 (SMD) with a 95% confidence interval of −22.58 to −16.19 (*p* < 0.00001), indicating strong statistical significance. It confirms that the effect sizes mostly assume negative values and are less than those of the control group. Consequently, this favours the nanocarriers over free cisplatin treatment. Specifically, it confirms that the effect sizes of the tumour volume in the nanocarriers mostly assume negative values compared to the control group. Thus, the tumour volume values in the nanocarriers are lower than those in the free cisplatin treatment group. The results depicted by the graphs displayed a significant difference (*p* < 0.00001) between the nanocarriers and free cisplatin as shown in Fig. 7b and c.

#### 3.2.4 The highest Pt concentration was in the tumours treated with nanocarrier -mediated cisplatin

**Fig. 8:**
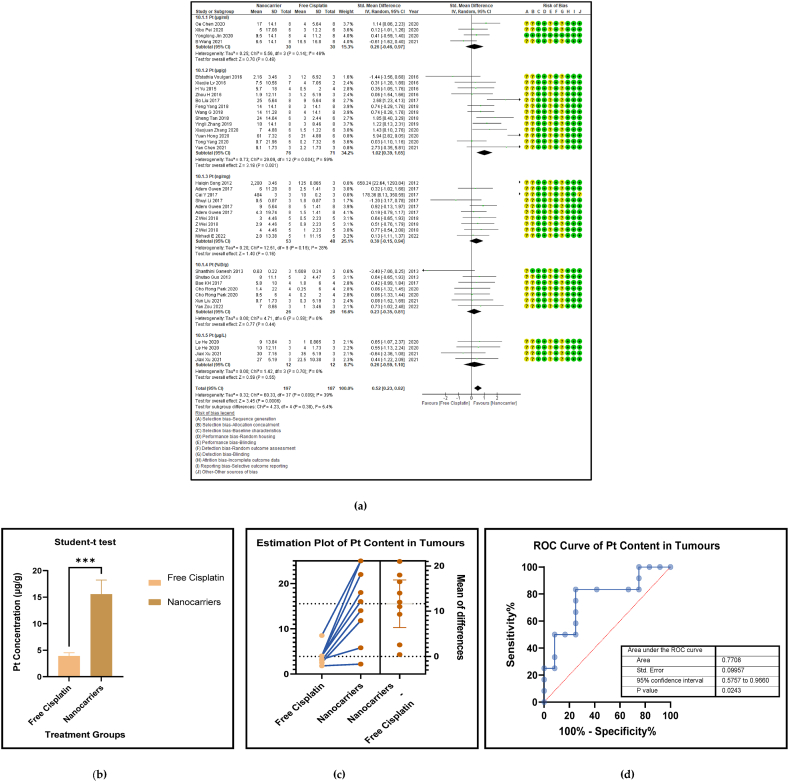
(a): Forest plot, (b) paired *t*-test as a bar graph (c) estimation plot and (d) ROC curve of the Pt content in the tumours in nanocarrier-mediated cisplatin delivery compared with free cisplatin treatment.

High bioaccumulation of any anticancer drug within the malignant tumours is what determines the bioactivity of that therapeutic formulation. The maximum Pt accumulation is achieved with the most potent anticancer formulation which is a measure of tumour attenuation despite high toxicity. Fig. 8 (a, b, c, and d) shows the forest plot, paired t-test-depicting bar graph, an estimation plot, and a ROC curve for the biodistribution of Pt in tumour tissue of the mouse models at sacrifice in the nanocarrier-mediated cisplatin delivery compared with free cisplatin treatment. The data revealed that the use of nanocarrier-mediated cisplatin led to a significantly higher tumour Pt concentration compared to free cisplatin treatment, with an overall pooled effect size of 3.88 (MD) and a 95% confidence interval of 2.89–4.87 (*p* < 0.00001), which indicated strong statistical significance. The Pt biodistribution in the tumours was calculated separately in four subgroups based on the units of cisplatin concentration shown in Table 5. The paired *t*-test on the results of the Pt tissue concentration in the tumours in (μg/g) participant data have indicated a significantly high Pt concentration in the nanocarrier group. Fig. 8c also reaffirmed the results obtained by the *t*-test. The tumour Pt content data in the nanocarriers mostly take positive values compared to the control group and thus the nanocarrier Pt concentration values were higher than those given free cisplatin treatment.

#### 3.2.5 The biodistribution of platinum in the vital organs treated with nanocarriers had a significant decrease in kidneys, and significant increases in the lungs and spleen

**Fig. 9:**
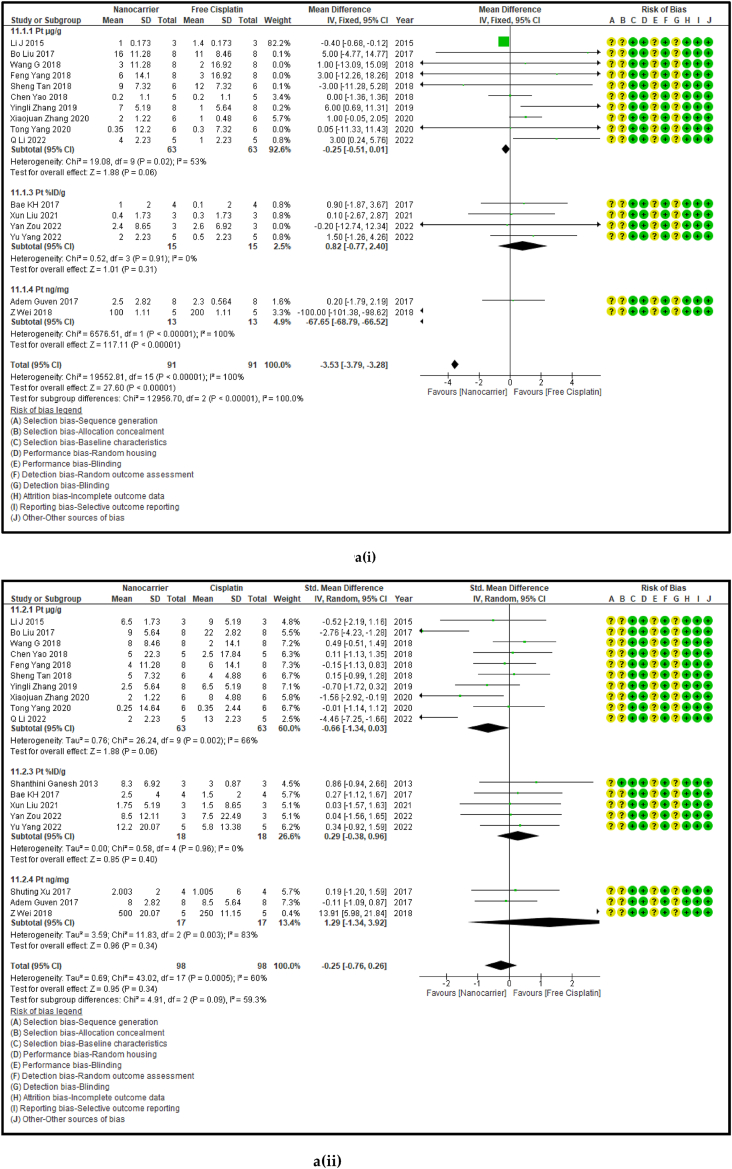

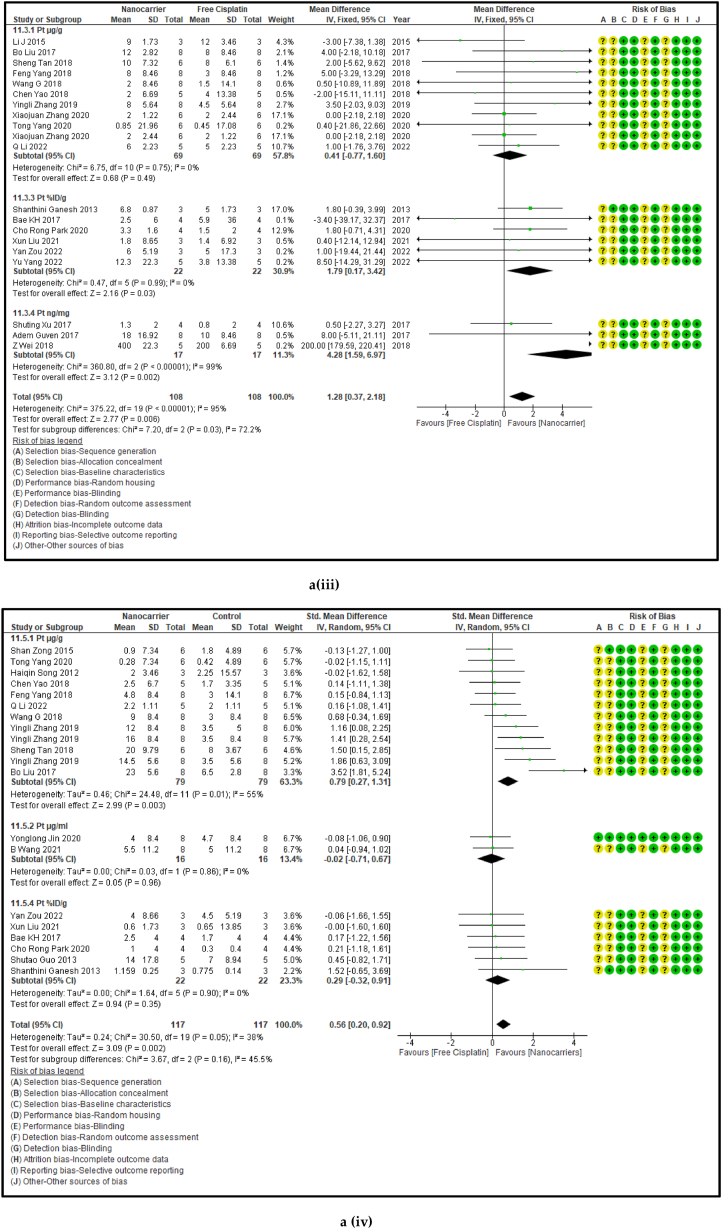

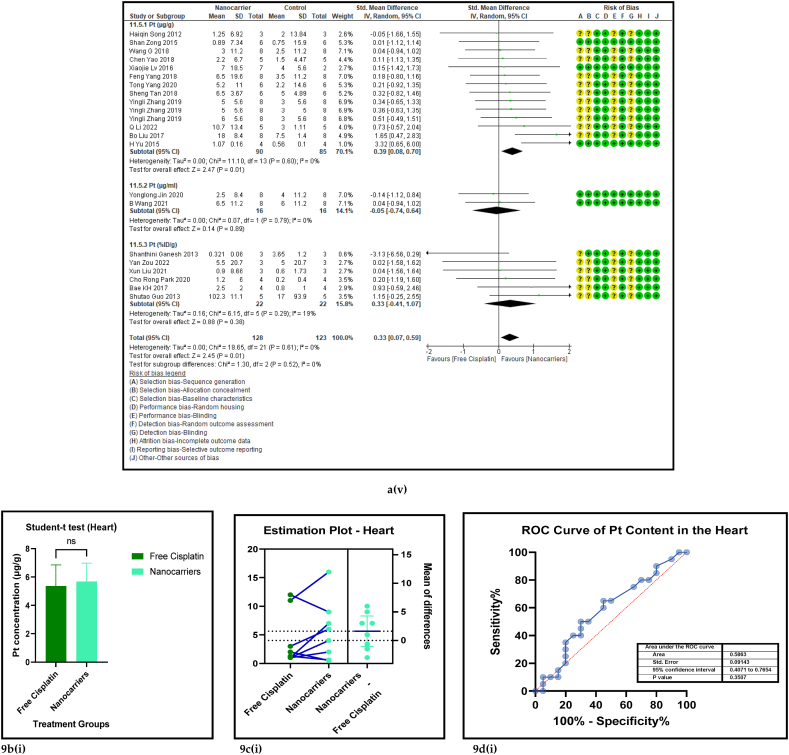

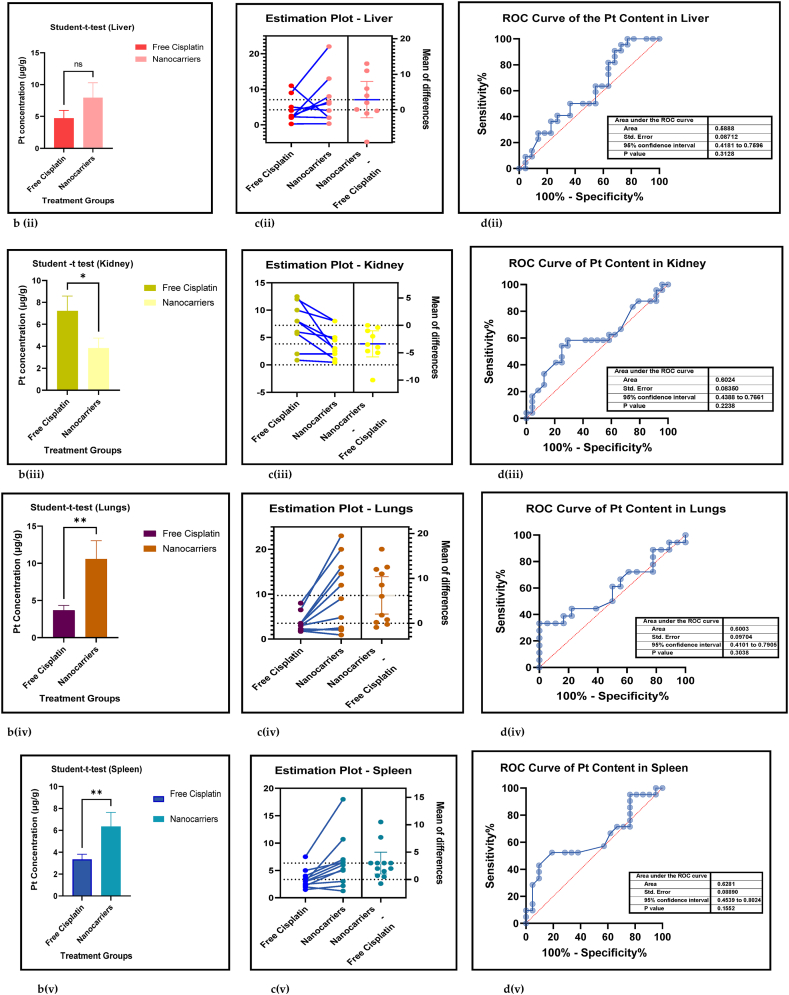
(a): Forest plots of Pt concentration in the vital organs, (b) paired t-test-depicting bar graphs, and (c) estimation plots and (d) ROC curves of the Pt biodistribution in the vital organs, (i) heart, (ii) liver, (iii) kidney, (iv) lungs, and (v) spleen, given free cisplatin compared with the nanocarrier-mediated cisplatin treatment.

The amount of Pt that has accumulated in the vital organs is a measure of biosafety of the given drug, which in this case is cisplatin. A low Pt biodistribution is preferred in the vital organs which must be shielded against its high toxicity. Fig. 9 [(ai-v) - (di-v)] shows the forest plots, paired t-test-depicting bar graphs, estimation plots and the ROC curves for the biodistribution of Pt in the (i) heart, (ii) liver, (iii) kidney, (iv) lungs, and (v) spleen respectively in the mouse models at sacrifice in nanocarrier-mediated cisplatin group compared with free cisplatin treated group. The data-analysis revealed that the use of nanocarrier-mediated cisplatin led to a significantly lower Pt content in the kidneys (probably because it was being excreted) and a non-significantly high Pt content in the liver (probably because the Pt is carried to the liver for detoxification) and heart (Pt is carried to the heart in the systemic circulation) and significant increases in the lungs (*via* the pulmonary circulation) and the spleen (for the mobilization of immune cells and overall immunity) compared to free cisplatin treatment. The paired t-tests revealed a significant difference in the Pt concentration in the kidneys, lungs and spleen between the control and the intervention groups [9b (iii-v)] and the estimation plots [Fig. 9c (iii-v)] also have reaffirmed the results produced by the *t*-test.

#### 3.2.6 Nanocarrier-mediated cisplatin treatment suppressed severe loss of body weight in xenograft mouse models of cancer

**Fig. 10:**
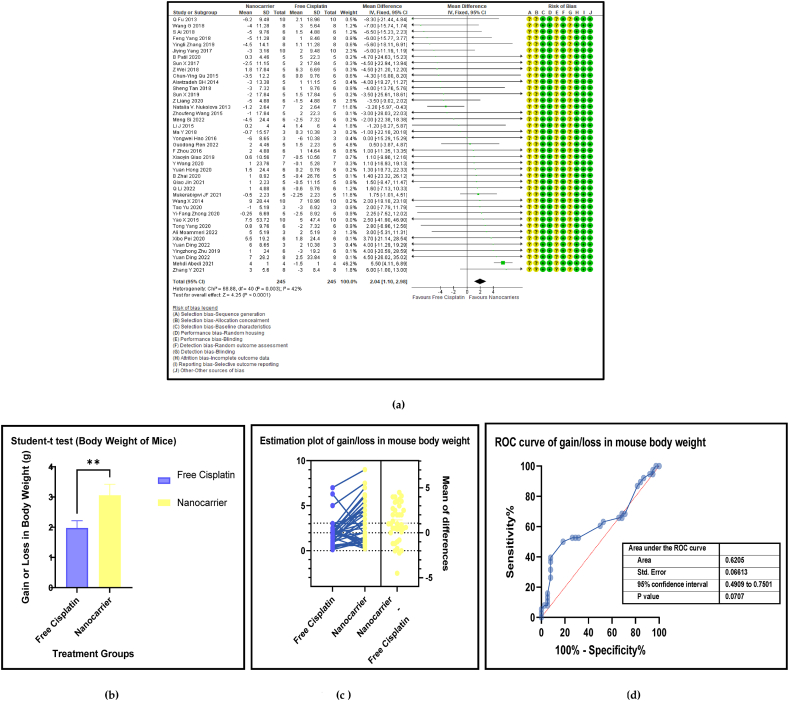
(a): Forest plot of gain or loss in body weight (g), (b) Paired t-test-depicting bar graph, (c) an estimation plot, and (d) ROC curve of the gain or loss of body weight in free cisplatin treatment compared with nanocarrier-mediated cisplatin.

The loss of body weight is a characteristic feature which is associated with cisplatin treatment, also documented as a prominent adverse effect of cisplatin that may induce low assimilation of nutrients in the intestines coupled with frequent vomiting and diarrhoea. Fig. 10 (a, b, c, and d) shows the forest plot, paired t-test-depicting bar graph, estimation plot and the ROC curve for the gain or loss of body weight at sacrifice in the mouse models treated with nanocarrier-mediated cisplatin compared with free cisplatin treatment. The overall pooled effect size was 2.04 (SMD) and a 95% confidence interval of 1.10–2.98 (*p* < 0.001), indicating strong statistical significance. The bar graph depicting the results of the paired *t*-test displayed a significant gain in body weight in the nanocarrier group (calculated as the body weight just before the treatment commenced subtracted from the final body weight at euthanasia). It confirms that the nanocarrier effect sizes mostly took positive values and were higher than those of the control group, and thus, consequently indicated a gain in the body weight compared to the free cisplatin treated group that mostly showed a marked loss in the body weight.

#### 3.2.7 Other haematological parameters also confirm the biosafety of nanocarrier-mediated cisplatin for attenuating cancer

**Fig. 11:**
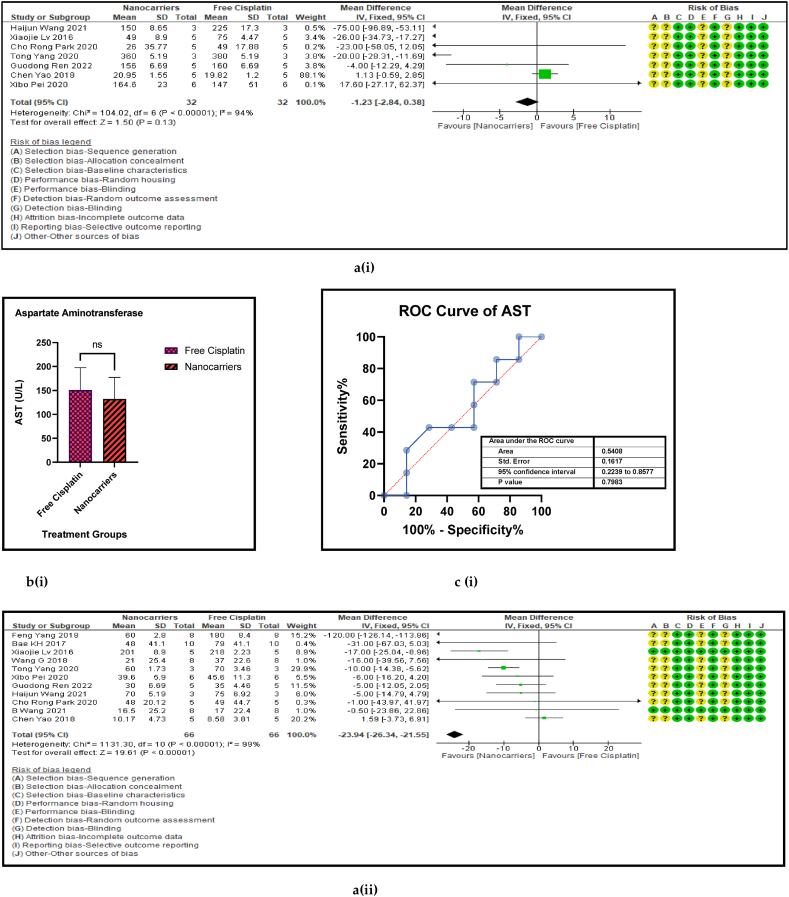

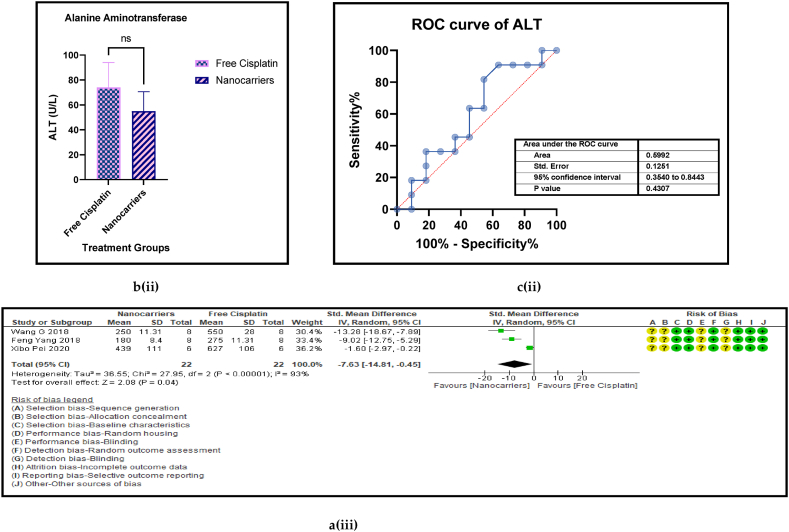

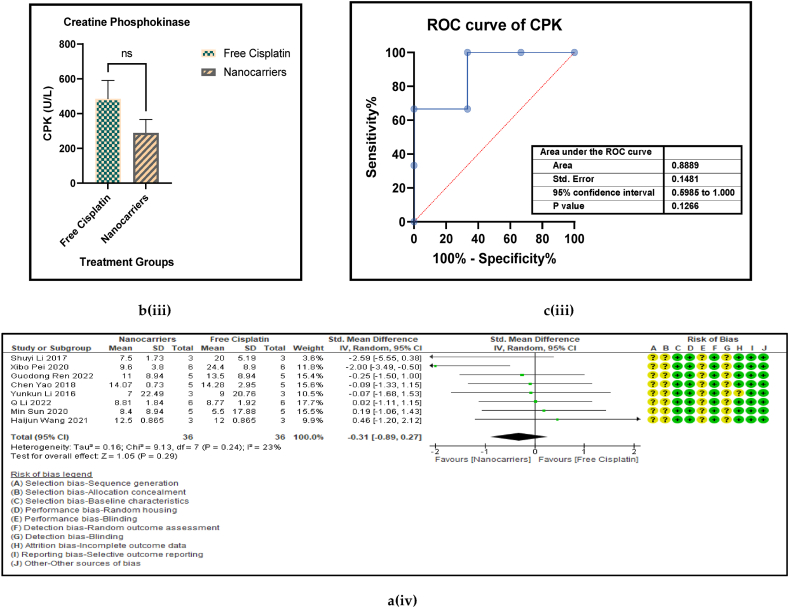

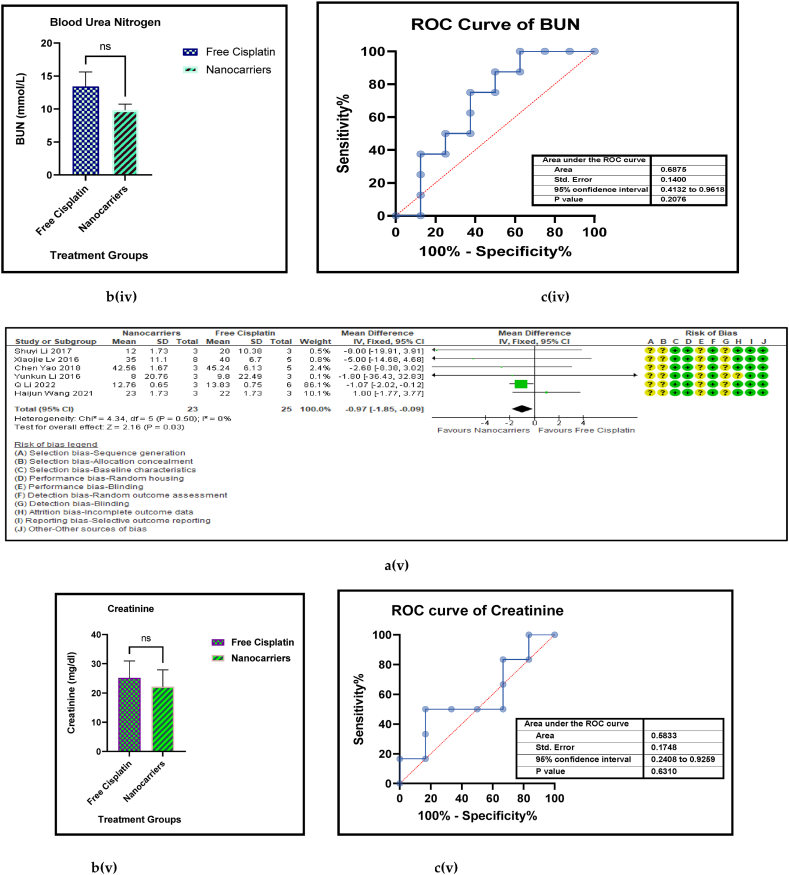
(a): Forest plots (b) paired t-test-depicting bar graphs and (c) ROC curves of the haematological parameters of further biosafety in free cisplatin treatment compared with nanocarrier-mediated cisplatin.

Haematological liver (AST, and ALT) and renal (BUN, and Creatinine) and both hepatic and renal (CPK) biomarkers have served as routine indicators of biosafety of any treatment given at the clinic when anticancer drugs are administered to cancer patients. Fig. 11 [ a, b, c, and d (i to v)] shows the forest plot, paired t-test-depicting bar graph, an estimation plot and the ROC curve respectively for the haematological parameters [aspartate aminotransferase (AST), alanine transaminase (ALT), blood urea nitrogen (BUN), creatinine, and creatine phosphokinase (CPK)] in the mouse models treated with nanocarrier-mediated cisplatin compared to the free cisplatin treatment. The forest plots displayed significant elevation in ALT and Serum creatinine in the nanocarrier group. All these haematological parameters were reported occasionally by a small number of the included studies. They are also inflammatory biomarkers that indicate toxicity in the liver and the kidneys. However, all these haematological indices were non-significantly suppressed in the nanocarrier - cisplatin treated mice (Table 8), indicating the efficiency of the nanocarriers in attenuating the high toxicity caused by cisplatin in routine treatment for cancers.

**Table 4:** Summary statistics for the tumour volume between free cisplatin delivery compared with the nanocarriers.

OUTCOME	STUDIES	# Mice	SMD	95% CI	Z SCORE	*P* < *0.05*	TAU^2^	I^2^%	Chi SQ	DF	*P* < *0.05*	*STUDENT t-TEST p VALUE*
**Overall Tumour Volume**	**68**	**891**	**−21.66**	**[-24.58, -18.74]**	**14.52**	***0.00001***	**77.82**	**93**	**974.13**	**73**	***0.00001***	***0.0001***

**Table 5:** Summary statistics for the Pt content in the tumours between the free cisplatin (control) group compared with the nanocarrier (treatment) group.

OUTCOME	STUDIES	# Mice	SMD	95% CI	Z SCORE	*P* < *0.05*	TAU^2^	I^2^%	Chi SQ	DF	*P* < *0.05*	*STUDENT t-TEST p VALUE*
Subgroup 1 Pt μg/ml	4	60	0.26	−0.46, 0.97	0.7	*0.48*	0.25	46	5.56	3	*0.14*	
Subgroup 2 Pt μg/g	13	147	1.02	0.39, 1.65	3.18	*0.001*	0.73	59	29.09	12	*0.004*	
Subgroup 3 Pt ng/mg	7	101	0.39	−0.15, 0.94	1.40	*0.16*	0.2	28	12.51	9	*0.19*	
Subgroup 4 Pt %ID/g	6	52	0.23	−0.35, 0.81	0.77	*0.44*	0.0	0	4.71	6	*0.58*	
Subgroup 5 Pt μg/L	2	24	0.26	−0.59, 1.10	1.42	*0.55*	0.3	0	1.42	3	*0.70*	
Overall Pt concentration in the tumours	32	384	0.52	0.23, 0.82	3.45	*0.0006*	0.32	39	60.33	37	*0.009*	*0.0178*

**Table 6:** Summary statistics for the Pt biodistribution in the vital organs between the free cisplatin (control) group compared with the nanocarrier (treatment) group.

OUTCOME	STUDIES	# Mice	SMD/MD	95% CI	Z SCORE	*P* < *0.05*	TAU^2^	I^2^%	Chi SQ	DF	*P* < *0.05*	*STUDENT t-TEST p VALUE*
9a (i) Heart												
Subgroup 1 Pt μg/g	10	126	−0.25	−0.51, 0.01	1.88	*0.06*	0.0	53	19.08	9	*0.02*	
Subgroup 2 Pt %ID/g	4	30	0.82	−0.77, 2.40	1.01	*0.31*	0.0	0	0.52	3	*0.91*	
Subgroup 3 Pt ng/mg	2	26	−67.65	−68.79, −66.52	117.11	*0.00001*	0.0	100	6571.56	1	*0.00001*	
Overall Pt concentration in the heart	16	182	−3.53	−3.79, −3.28	27.60	*0.00001*	0.0	100	19552.81	15	*0.00001*	*0.7154*
9a (ii) Liver												
Subgroup 1 Pt μg/g	10	126	−0.66	−1.34, 0.03	1.88	*0.06*	0.76	66	26.24	9	*0.002*	
Subgroup 2 Pt %ID/g	6	44	0.33	−0.28, 0.93	1.06	*0.29*	0.0	0.0	0.65	5	*0.99*	
Subgroup 3 Pt ng/mg	3	34	1.29	−1.34, 3.92	0.96	*0.34*	3.59	83	11.83	2	*0.003*	
Overall Pt concentration in the liver	19	204	−0.25	−0.76, 0.26	0.83	*0.41*	0.66	59	43.91	18	*0.0006*	*0.2359*
9a (iii) Kidneys												
Subgroup 1 Pt μg/g	10	138	0.41	−0.77, 1.60	0.68	*0.49*	0.0	0.0	6.75	10	*0.75*	
Subgroup 2 Pt %ID/g	6	44	1.79	0.17, 3.42	2.16	*0.03*	0.0	0.0	0.47	5	*0.99*	
Subgroup 3 Pt ng/mg	3	34	4.28	1.59, 6.97	3.12	*0.002*	0.0	99	360.80	2	*0.00001*	
Overall Pt concentration in the kidneys	19	216	1.28	0.37, 2.18	2.77	*0.006*	0.0	95	375.22	19	*0.00001*	*0.0109*
9a (iv) Lungs												
Subgroup 1 Pt μg/g	9	146	0.88	0.34, 1.43	3.19	*0.001*	0.45	55	21.98	10	*0.02*	
Subgroup 2 Pt μg/ml	2	16	−0.02	−0.71, 0.64	0.05	*0.96*	0.0	0.0	0.03	1	*0.86*	
Subgroup 3 Pt %ID/g	6	44	0.29	−0.32, 0.91	0.94	*0.35*	0.0	0.0	1.64	5	*0.90*	
Overall Pt concentration in the lungs	17	206	0.65	0.26, 1.03	3.44	*0.0006*	0.26	38	25.75	16	*0.06*	*0.0056*
9a (v) Spleen												
Subgroup 1 Pt μg/g	12	175	0.39	0.08, 0.70	2.47	*0.01*	0.0	0.0	11.10	13	*0.60*	
Subgroup 2 Pt μg/ml	2	16	−0.05	−0.74,0.64	0.14	*0.89*	0.0	0.0	0.07	1	*0.79*	
Subgroup 3 Pt %ID/g	6	44	0.33	−0.41, 1.07	0.88	*0.38*	0.16	19	6.15	5	*0.29*	
Overall Pt concentration in the spleen	20	235	0.39	0.11, 0.67	2.70	*0.007*	0.0	0.0	17.26	19	*0.57*	*0.0067*

**Table 7:** Summary statistics of the gain or loss in mouse body weight between the free cisplatin treatment compared with nanocarrier -cisplatin.

OUTCOME	STUDIES	# Mice	SMD	95% CI	Z SCORE	*P* < *0.05*	TAU^2^	I^2^%	Chi SQ	DF	*P* < *0.05*	*STUDENT t-TEST p VALUE*
Overall gain or loss in mouse body weight	40	490	2.04	1.10, 2.98	4.25	*0.0001*	0.0	42	68.88	40	*0.003*	*0.0034*

**Table 8:** Summary statistics of some haematological parameters of biosafety in nanocarriers delivering cisplatin to tumours in mice.

OUTCOME	STUDIES	# Mice	SMD/MD	95% CI	Z SCORE	*P* < *0.05*	TAU^2^	I^2^%	Chi SQ	DF	*P* < *0.05*	*STUDENT t-TEST p VALUE*
AST	6	54	−1.18	−2.80, 0.43	1.44	*0.15*	0.0	95	102.54	5	*0.00001*	*0.1471*
ALT	11	132	−23.94	−26.34, −21.55	19.61	*0.00001*	0.0	99	1131.30	10	*0.00001*	*0.0987*
Creatine phosphokinase (CPK)	3	44	−7.63	−14.81, −0.45	2.08	*0.04*	36.55	93	27.95	2	*0.00001*	*0.0818*
BUN	8	72	−0.31	−0.89, 0.27	1.05	*0.29*	0.16	23	9.13	7	*0.24*	*0.1619*
Creatinine	5	35	−0.94	−1.82, −0.05	2.08	*0.04*	0.0	0	3.67	4	*0.45*	*0.0733*

#### 3.2.8 Certainty of evidence and high heterogeneity was observed among the data extracted from the included studies

This study comprised of widely varied data within the seven primary outcomes that have been evaluated. Hence, there was high heterogeneity among the data extracted from the included studies. In addition to the calculated Chi squared distribution and the I squared metric; the Q statistic was also computed. A meta-analysis by virtue is a compilation of diverse experimental data that have similar characteristics and content that had been carried out with similar protocols. The outcome data without outliers was considered as valid data. There are 3 ways in which heterogeneity between the included studies can be analysed, as per the Cochrane guidelines. They are (1) visual inspection of forest plots based on the individual effect sizes, (2) the Q statistic (Table 9), and (3) the I squared statistic. According to the I squared statistic, the included studies in this meta-analysis have high heterogeneity because a I squared metric greater than 70% is considered having high heterogeneity. It is also important to examine the magnitude and direction of its effect. A random effects model was used in most cases, and the direction of the results was definitely favouring the nanocarriers indicating credibility of data and certainty of evidence in the overall study (Table 8).

**Table 9:** Computation of the Q statistic.

PRIMARY OUTCOME	HIGHEST MEAN (A)	MEAN CLOSEST TO HIGHEST MEAN (B)	DIFFERENCE BETWEEN A AND B (C)	RANGE	DIVIDE (C) BY RANGE	Q STATISTIC	CRITICAL VALUE OF Q AT *P* < 0.05	Df = n-1	Q STATISTIC IS LESS THAN CRITICAL VALUE	ACCEPT/REJECT NULL HYPOTHESIS (MAX VALUE IS NOT AN OUTLIER
Cancer cell viability %	75	73.5	1.5	75–2	1.5/73	0.02	2.858	45	Less	Not an outlier
IC50	175	120	55	175–0.003	55/174.997	0.314	2.913	25	Less	Not an outlier
Tumour volume	3000	1500	2000	3000–2.79	2000/2997.1	0.66	2.829	60	Less	Not an outlier
Pt content in tumours	2200	484	1716	2200–0.5	1716/2199.5	0.78	3.055	13	Less	Not an outlier
Pt content in heart	100	16	84	100–0.2	84/99.8	0.84	3.014	15	Less	Not an outlier
In liver	500	13.8	486.2	500–0.2	486.2/499.8	0.97	2.984	17	Less	Not an outlier
In kidneys	400	18	382	400–0.85	382/399.15	0.95	2.984	17	Less	Not an outlier
In lungs	23	20	3	23–0.28	3/22.72	0.13	3.055	13	Less	Not an outlier
In spleen	102.3	18	84.3	102.3–0.321	84.3/101.979	0.82	2.998	16	Less	Not an outlier
AST	360	164	196	360–20.95	196/339.05	0.57	3.460	6	Less	Not an outlier
ALT	201	70	131	201–10.17	131/190.83	0.68	3.151	10	Less	Not an outlier
CPK	439	250	189	439–180	189/259	0.72	6.085	2	Less	Not an outlier
BUN	14.07	12.5	1.57	14.07–7	1.57/7.07	0.22	3.344	7	Less	Not an outlier
Creatinine	42.56	35	7.56	42.56–8	7.56/34.56	0.21	3.635	5	Less	Not an outlier
Gain/loss in body weight	9	7.5	1.5	9— (−) 6.2	1.5/15.2	0.098	2.858	40	Less	Not an outlier

#### 3.2.9 Publication bias

**Fig. 12:**
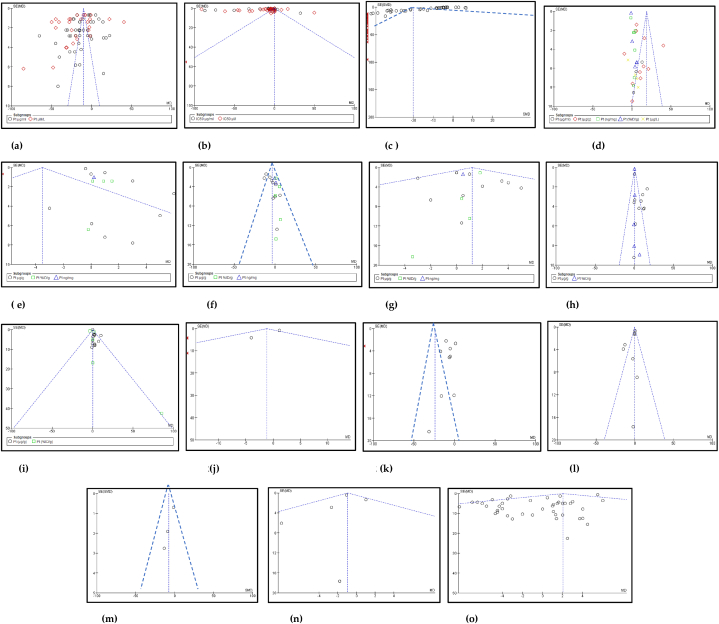
(a–h): The funnel plots that display minimalized publication bias in (a) cancer cell viability %, (b) IC50 of the cancer cells, (c) tumour volume, (d) Pt distribution in tumour tissue, (e) Pt distribution in the heart, (f) Pt distribution in the liver, (g) Pt distribution in the kidney, (h) Pt distribution in lungs, (i) Pt distribution in spleen, (j) AST, (k) ALT, (l) CPK, (m) BUN, (n) creatinine, (o) gain or loss in body weight of the animals, in nanocarrier-mediated cisplatin compared with free cisplatin treatment in the included studies.

**The funnel plots are tools which measure publication bias in each individual study**Fig. 12-(a-h) display the funnel plots of the included studies for each outcome, with larger sample sizes plotted near the top and smaller sample sizes plotted near the bottom. The distribution of studies is symmetrical around the midline, in most of the primary outcomes that were tabulated indicating the absence of publication bias. In the funnel plot of cancer cell viability %, a total of 47 publications were included and they were spread bilaterally around the midline towards the top, indicating the sample sizes were large because it had 660 mice (Fig. 12a). In the funnel plot of IC50, there were 26 studies that were also spread bilaterally towards the top comprising of 398 participants (Fig. 12b). The tumour volume included 68 studies which had 891 mice, and as such most of the samples were clustered at the top and along the right side of the funnel plot, although not spread evenly around the midline (Fig. 12c). The Pt concentration in tumour tissue included 32 studies with 384 mice which were spread symmetrically closer to the midline (Fig. 12d). The Pt biodistribution in the heart had 16 studies and 182 mice, the liver had 19 studies with 204 mice, and the kidneys included 19 studies with 216 mice, the lungs had 17 studies with 206 mice, and the spleen had 20 studies with 235 mice that had the studies spread bilaterally in narrow funnel plots closer to the midline (Fig. 12e–i). The AST, ALT, CPK, BUN and Creatinine each had 6 studies with 54 animals, 10 studies with 122 animals, 3 studies with 44 animals, 8 studies with 72 animals and 6 studies with 41 animals respectively (Fig. 12 j-n). The gain or loss in body weight comprised 40 included studies with 490 mice that were spread bilaterally in the middle of the funnel plot, although skewed more to the left side with more samples having a negative SMD (Fig. 12o) that indicates having small sample sizes. No publication bias was visible in the following primary outcomes: cancer cell viability %, Pt biodistribution in the tumours, liver, lungs and spleen, ALT and the gain or loss in body weight in the mice. These conventional outcomes had narrower funnel plots spreading bilaterally from the top to bottom almost evenly. Large sample sizes having high SMD or MD were seen in the funnel plots of the Pt biodistribution in the kidneys (Fig. 12g), IC50 and tumour volume (Fig. 12 b and c), and the gain or loss in body weight (Fig. 12o).

### 3.3 Risk of bias in included studies

**Fig. 13:**
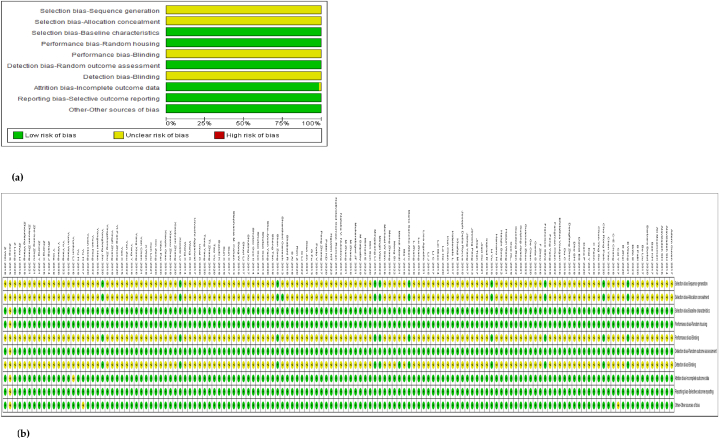
**(a): The risk of bias summary of the included studies. (b): The judgement of risk of bias for each study using the** SYRCLE's **reporting of bias tool which is appropriate for animal studies.**

The risk of bias assessment (Fig. 13a and b) was carried out using the built-in application in the RevMan 5.4 software. In almost all the included studies, selection bias was minimal because the animals were randomly distributed among groups. The risk of bias analysis for this study was performed using the parameters of the SYRCLE's tool which is more relevant to animal studies and includes 10 parameters. The performance and detection bias had an unclear risk due to insufficient information regarding blinding procedures. Attrition bias was minimal because there were almost nil premature deaths reported in the sampling cohorts. There was reporting bias to some extent because negative results were hardly reported. The sample size (animal number per sample) varied between n = 3–10 and it was seen as another potential source of bias.

### 3.4 Multiple regression analysis

**Fig. 14:**
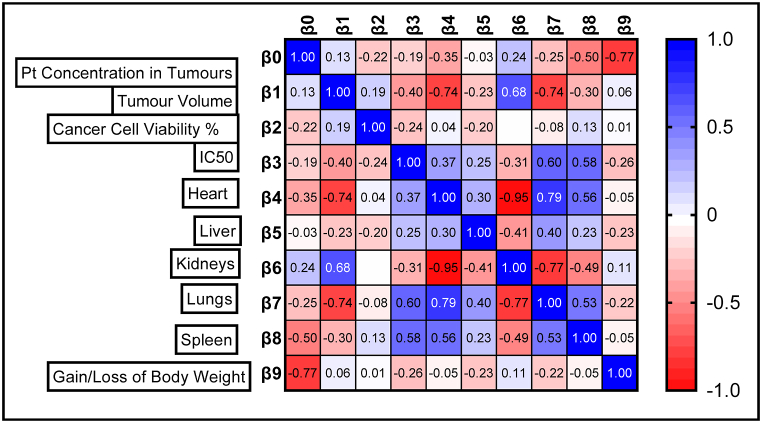
Multiple regression analysis between the independent variable (Pt concentration in tumours) with the dependent variables which are the other primary outcomes. β0 is the intercept of the linear XY graph when X = 0, β1 is tumour volume, β2 is cancer cell viability, β3 is IC50, β4 is biodistribution of Pt in heart, β5 is biodistribution of Pt in Liver, β6 is biodistribution of Pt in kidney and β7 is Pt in the lungs, β8 is Pt in the spleen, and β9 is the gain or loss in body weight of the animals.

Multiple regression is a statistical technique that can be used to analyse the relationship between a single continuous dependent variable and several independent variables [142]. In all respects, this is the same analysis as a *t*-test, with the same assumptions and same results. The use of the beta coefficient allows direct comparisons between independent variables to determine which has the most influence on the dependent variable. A positive coefficient indicates that as the value of the independent variable increases, the mean of the dependent variable also tends to increase. A negative coefficient suggests that as the independent variable increases, the dependent variable tends to decrease. A positive beta coefficient indicates a positive relationship, and a negative coefficient indicates a negative relationship. Frequently researchers will select a sample size and decision rule to ensure that beta is 0.20 or less (or having an equivalent power of 0.80 or more). Some researchers prefer to ensure that the beta level is 0.10 or less. A standardized beta coefficient compares the strength of the effect of each individual independent variable to the dependent variable. The higher the absolute value of the beta coefficient, the stronger the effect (Fig. 14) [142].

## 4 Discussion

There is a plethora of recent to old publications on nanotechnological applications that offer customised cancer treatment with cisplatin. They delivered cisplatin by itself or as a conjugate for better synergism [143]. However, all of it has not been reviewed in this study as it is beyond the scope of this meta-analysis. The present study consists of a cross-sectional analysis comprising of 94 research studies performed on xenograft mouse models within the last decade. Those studies utilized a spectrum of varied nanoparticular systems that were evaluated for the benefit of nanocarrier-mediated cancer therapy with cisplatin. The major drawback of free cisplatin therapy on its own is severe toxicity that debilitates multiple organ systems [144]. The adverse effects arose from unspecific drug targeting [145], multi-drug resistance [146] and dysregulated metabolism [147]. A recent advance made in chemotherapy is the introduction of smart nanoparticles as a drug delivery platform to address the deficiencies in conventional anticancer treatment [148]. They achieve target-specific precise drug delivery triggered by various stimuli that impact and modulate the tumour microenvironment (TME) with a higher success rate while utilizing the existing curatives [149]. The smart nanoparticles combined with artificial intelligence have raised the cancer-specific therapies to the next level of precision medicine, including cisplatin that is set to revolutionize the nanotechnology-based cancer treatment with testing the age-old drugs [150]. These remedial measures largely encompass the external and internal stimuli or exogenous and endogenous physical parameters that influence the drug pharmacokinetics [151]. The parameters included pH, temperature, enzyme activity of the TME with nanoparticle size, shape, surface texture, encapsulation efficiency, sustained rate of drug release, uploaded drug concentration, biodegradability, longer plasma circulatory time and drug elimination rate from the liver and kidneys [[151], [152], [153]]. As such, these characteristics contribute to the dynamic capabilities of nanocarrier-mediated cisplatin treatment.

**Fig. 15:**
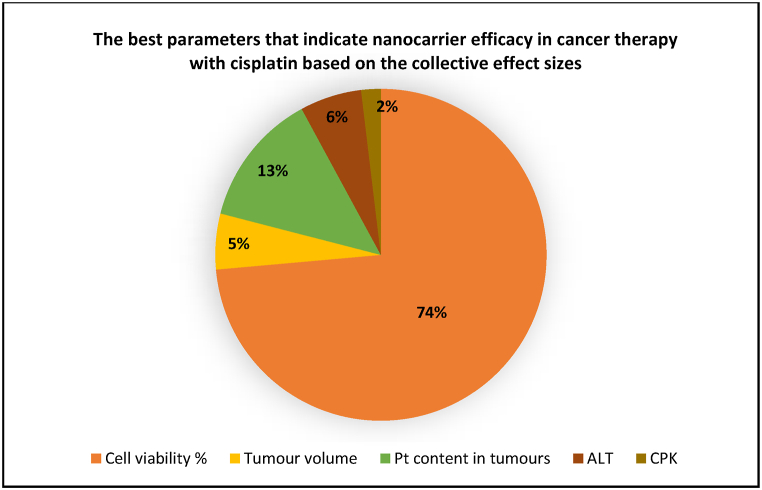
A pie chart depicting the best of the primary outcomes that were investigated in this study, based upon the overall effect sizes.

Therefore, as the prevailing published mouse research studies have permitted, the present study has assessed seven different categories of preclinical primary outcomes from the reported data. They evaluate the advantages presented by nanoparticle-entrapped cisplatin treatment, for several different types of inducible cancers. The 7 categories are the (1) cancer cell viability percentage, (2) IC50 of the cancer cells, (3) tumour volume regression (4) Pt biodistribution concentration in the tumours, (5) Pt biodistribution concentration in the vital organs; (i) heart, (ii) liver, (iii) kidneys, (iv) lungs, (v) spleen, (6) the gain or loss in body weight, and (7) the haematological/inflammatory parameters that indicate biosafety of the nanocarriers; (i) AST, (ii) ALT, (iii) CPK, (iv) BUN, and (v) Creatinine. The data from each of these outcomes present evidence of the superiority of the nanocarriers over free drug treatment. The best parameters of the 7 outcomes are graded based on the effect sizes obtained from the forest plots, from highest to the lowest in a pie chart shown below (Fig. 15).

The superiority of nanocarrier-encapsulated cisplatin over free drug therapy in cancer-bearing preclinical mouse models is reaffirmed in this meta-analysis, which is the first of its kind, to the best of our knowledge. The protocol for this study was registered in the PROSPERO database in December 2022 (Available from: https://www.crd.york.ac.uk/prospero/CRD42023369333). The major finding of this study which utilized 94 included studies was that irrespective of the different nanocarrier types and the methods of synthesis, the end result was that nanocarrier – mediated cisplatin delivery conformed to a successful anti-cancer therapeutic modality. It is capable of overcoming the varied impediments a clinician faces in prescribing cisplatin in the clinic. The translational and the theragnostic efficiency of nanocarrier-delivered cisplatin from the bench to bedside is supported by our analytical evidence as follows; precise tumour cytotoxicity (*p* < 0.00001) displayed by lower cell viability % in the cancer cells *in vitro* (Fig. 5a–d and Table 2), lower IC50 in cancer cells (*p* < 0.0001), (Fig. 6a–d and Table 3), remarkable regression in the tumour volume (*p* < 0.00001), (Fig. 7a–d and Table 4), the accumulation of higher Pt concentration in tumours (*p* < 0.0006), (Fig. 8a–d and Table 5), significant Pt biodistribution in the kidneys (p < 0.006), lungs (p < 0.0006) and spleen *(p* = 0.007), (Fig. 9a–d, i-v and Table 6), the haematological and inflammatory indicators that exhibit the biosafety of the nanocarriers (Fig. 10 a-e, i-v and Table 8) which had ALT at (p < 0.00001), CPK and creatinine at (p < 0.04), and minimal loss of body weight (*p* < 0.0001), (Fig. 10a–c and Table 7). These parameters were based on the Z-score computations which confirm the suitability of nanocarriers for enhancing cisplatin's bioactivity, specifically in xenografted mouse models. These observations are further supported by the parametric statistical test-the paired t-distribution values and the estimation plots which show significant differences between the control and intervention groups for the primary outcomes as shown by Fig. 5, Fig. 6, Fig. 7, Fig. 8, Fig. 9, Fig. 10b and Fig. 5, Fig. 6, Fig. 7, Fig. 8, Fig. 9, Fig. 10 respectively. The multiple regression analysis displayed weaker associations between tumour volume, IC50, cell viability and the biodistribution of Pt in the heart, liver, and kidneys (Fig. 14). The funnel plots (Fig. 12a–h) that display the number of included studies in each of the outcomes utilized within the given time range (spanning the years 2012–2023) indicate there is no significant publication bias. The studies with higher standard mean difference (SMD) or the mean difference (MD) are present at the top of the graph while those with lower SMD or MD are dispersed towards the bottom.

The nanocarriers can be broadly categorized to lipid-based, polymeric, and inorganic nanocarriers which are endowed with distinctive advantages that favour their utility as a drug delivery platform [154]. This study includes 11 lipid-based, 28 polymeric, 12 inorganic nanocarriers with two more additional groups of 25 lipid-based and polymeric and 12 inorganic and polymeric groups (Fig. 2a). Hence this meta-analysis includes a wide variety of nanocarriers of which the varying characteristics contribute to enhancing the nanocarrier-based drug delivery efficiency, thus validating the applicability of the outcomes produced by this study. Lipid-based nanocarriers possess the advantage of easy penetration, quick accessibility, and diffusion from lymphatics to the blood circulation [155]. They are mostly poly-ethylene-glycolate (PEGylated) surfactants that form solid nanoparticles which improve drug solubility and bioavailability, mostly in the central nervous system. The polymeric nanoparticles are colloidal solids that allow for the accumulation of poorly soluble drugs in tumours *via* the oral administration route, and composed of common polysaccharides such as chitosan, collagen etc, conferring high bioavailability, quick penetration into the blood circulation and being easily biodegradable [155]. Inorganic nanocarriers allow for reduced cytotoxicity of medicines, good biocompatibility, stimulating ROS generation and antibacterial action by being nontoxic, hydrophilic, and very stable, drug-delivery composites [154]. Further, this study includes pooled relevant data from 12 different xenograft mouse models, 19 different inducible cancer types and 18 different cancer cell lines, of which the multiplicity has increased its validity (Fig. 2b–d).

The use of nanotechnology for synergistic dual drug systems presents with biocompatibility, safety and efficient drug release which helps to additionally overcome chemoresistance [156]. The surface decorations/modifications on nanocarriers contribute to enhance biocompatibility [157]. A commonly used material for surface decorations having higher bioactivity is phospho-ethylene-glycol (PEG) and folic acid [158]. The PEG-functionalised nanocarriers had low cytotoxicity and less organ damage even after intravenous injections at a high dose of the drug in mice. PEG has been a successful modifier of the nanocarriers which influences the physicochemical and biopharmaceutical properties of the nanocarriers that confer enhanced therapeutic potential with less side effects [159,160]. The deficiency is having higher toxicity than normal, which also could be counteracted by tumour centric delivery which is an advantage of the nanocarriers [161]. The other benefits of nanocarrier use is having sustained release of the drugs and longer plasma circulatory time [162]. The nanoscale or size is a critical factor in getting the nanoparticles internalized [163]. The nano (nm) and micro (μm) sized carriers have been described in certain studies as being easily taken up by the macrophages [164]. The nanoparticles increase in size when protein molecules are adsorbed onto its surface and form aggregates that reduce the prospects of internalization [165]. The nanocarriers have shown distinctive *in vivo* behaviour as well, such as reduced reticuloendothelial exposure and passive targeting of tumours [166]. The drug releasing kinetics of nanocarriers is pH dependent [167] and impacted by electrostatic forces that exist between the non-ionic surfactants and the drug [168]. The ionization state of the physiological pH also influences the drug releasing rate [169]. Another aspect of nanocarrier use is its ability to dissociate in acidic pH, produced by repulsive forces that develop between the positively charged drugs and PEG chains [170,171]. The pH-sensitive prodrugs are utilized in targeted delivery to the tumours, particularly in acidic pH [172]. Similarly, glutathione -sensitive prodrugs also offer excellent anticancer activity [173]. Redox-responsive nanoparticles were found to release the encapsulated drug 4 times over than without glutathione [174]. The solution to the drug-resistant cancers has been combinatorial treatment [175] such as cisplatin with docetaxel or paclitaxel or Epirubicin-co-loaded nanovesicles, that displayed synergistic tumour inhibition confirmed by different cancer models including lung cancer [176]. The enhanced permeability and retention effect (EPR effect) is an established phenomenon in nanocarrier-mediated anticancer treatment, in which leaky blood vessels and poor lymphatic circulation within the tumour vasculature facilitates targeted tumour destruction [177]. The storage stability of the nanocarriers is an important aspect which affects the therapeutic potential of the drugs where high storage stability offers good protection from being disintegrated by the nucleases in circulating blood plasma [178]. When combining nanocarriers with prodrugs, they must cooperate with each other to produce better therapeutic effect [179]. The macromolecular prodrugs are the most common prodrugs utilized in nanomedicines [180]. Certain nanocarriers employ tumour targeted ligands that bind with most common receptors on tumour cells, to allow easy accumulation of chemotherapeutics in tumours [181]. Further, the position or point at which the prodrug is introduced, influences the stability and therapeutic efficacy of the nanovesicles [182], with sustained and preferential drug-release into endosomes or lysosomes in tumour cells without emptying its contents into the general blood stream which happens with the free drug administration [183,184].

The size of the nanocarrier is a critical measurement of its efficiency as a drug carrier which facilitates entry to the cells. The size of the nanocarriers in the included studies varied between 30 and 192 nm with a median value of 100 nm. The Australian nanotechnology network includes over 80 organizations, and the University of New South Wales has undertaken an ongoing study on the efficacy and precision nanomedicine based on the size of the nanocarriers for the treatment of childhood acute lymphoblastic leukemia which has a high rate of childhood mortality [185]. This ongoing study in its preliminary stage has reported higher anti-cancer efficiency with smaller sized nanocarriers than with the larger sizes. The spherical nanocarriers have the added advantage of having a larger surface area for imbibition and drug release.

In interpreting the forest plots, the vertical line is the line of no effect (the position at which there is no clear difference between the intervention group and the control group) [186]. The boxes show the effect estimates from the individual studies, while the diamond shows the pooled result [187]. The horizontal lines through the boxes illustrate the length of the confidence interval [188]. The longer the lines, the wider the confidential interval, the less reliable are the study results [188]. The width of the diamond serves the same purpose [189]. The cell viability % estimates are present on the left side of the mid vertical line of no effect which means the nanomedicine is favoured over the free drug treatment. That is, the outcome of interest (cell viability of *in vitro* cancer cell lines) was lower in the intervention group than in the free cisplatin control group. The forest plot estimates for IC50, tumour volume and the Pt content in tumour tissue and the kidneys, lungs and spleen are also located on the left side of the line of no effect, which indicates that those values are lower in the intervention group which is the nanocarrier cisplatin treatment. If the diamond touches the vertical line or if the 95% spans from positive to negative values, the overall (combined) result is not statistically significant [190]. It means that the overall outcome in the intervention group is much the same as in the control group [191]. The cell viability %, IC50, tumour volume, the Pt content in tumours, kidneys, lungs, spleen, ALT, and creatinine estimates display significant differences between the nanocarrier group and its control cohort. The biodistribution of Pt in the heart and liver does not show a significant difference between the intervention and control groups. The biodistribution of Pt in the kidneys is lower than that of the control group, which means that the nanocarriers have reduced accumulation of Pt in renal tissue, the kidneys being the major organs that excrete toxic substances and the kidneys and liver being the primary organs of detoxification. The Pt concentration in the liver appears on the right side of midline indicating that the nanocarriers are not favoured over the free drug control. It is possibly because the nanocarriers are efficiently internalized by endocytosis or receptor mediation, and thus accumulate more readily within the liver tissue for elimination through detoxification, thereby showing a higher Pt concentration in the liver. The *p* value indicates the level of statistical significance. The difference between the two groups was statistically significant if the diamond shape did not touch the line of no effect [191]. In that case, the *p* value is usually <0.05. The I^2^ indicates the level of heterogeneity [192]. It can take values from 0% to 100%. If I^2^ ≤ 50%, studies are considered having low heterogeneity, and a fixed effect model of meta-analysis can be used. If I^2^ > 50%, the heterogeneity is high, and a random effects model should be used for the meta-analysis [192]. The difference between homogeneity and heterogeneity therefore lies in the different approaches taken to calculate the pooled result [193]. The funnel plots displayed low publication bias, having larger sample sizes clustered towards the top of the graph while the lower sample sizes were found scattered more towards the bottom. A sensitivity and certainty of evidence is provided by a ROC curve that was produced for each outcome [194,195]. The Bonferroni correction was not applicable because in all the comparisons, the same control group was not utilized twice. For completeness to assess the study quality, the SYRCLE's checklist that is applicable to animal studies was used in this study [196]. However, the main drawback of the favourable results obtained from this meta-analysis is that it is questionable as to how effectively the outcome could be translated or related to personalized clinical cisplatin therapy to treat cancers in individual patients [197]. Although the mouse models are considered to provide experimental evidence that closely resembles human pharmacokinetics, considerable variability still exists between the preclinical and clinical cancer models [198]. Another disparity that exists between mouse studies and clinical trials is the duration and number of the treatment cycles, because the maximum chemotherapeutic cycles given to mice is limited to either 3 or 4 and the maximum life span of these animals post-commence the regimen is quite short, owing to the rapid loss of body weight in some that will require euthanasia under the ethical guidelines [199]. In cancer patients, the treatment cycles continue beyond the preclinical experimental duration, having a maximum survivability up to 5 years [200]. Another limitation of this meta-analysis is not having enough studies using the same nanocarrier for comparison which could be compared with, for several seem to be the first of this category. This study could be considered as the baseline to investigate further outcomes.

For future investigations, an evaluation of nanocarriers combined with immunotherapy is suggested because immunomodulation will take precedence over the largely non-effective chemoradiation therapy that produces higher toxicity in resolving carcinomatous disease. The modern advances in nanotechnology-based drug delivery comprise exploring new avenues in cancer therapy with tumoroids, 3D e-bioprinting and epigenetic manipulation. The importance is to overcome the debilitated immunity that allows for the development, persistence, and metastasis of the cancers, wholly attributed to chemoresistance leading to immune failure, which has now proven surmountable with the nanomedicine approach.

While there is improved efficacy of cisplatin therapy with nanocarriers, there are many limitations to the use of nanoparticles to deliver cisplatin treatment [12]. The toxicity is increased with the complexity of the nano formulations that allow accumulation of the drug in unwanted locations [12]. The non-uniform size, shape and structure could develop irregularities in cellular uptake warranting precise assessments between the nanocarrier drug loading and release rates [201]. Mass reproducibility, long-term storage and other issues pertaining to commercialized production could lead to difficulties in the manufacturing process [202]. It is simply not a case of encapsulating a mixture of drugs, but the identification and evaluation of the drug metrics and ensuing side effects that should meticulously align with the pharmacokinetics to suit the host's physiological and immunological attributes required for efficient metabolism [203]. There are many challenges the researchers face by lacking an in-depth understanding of the drug mechanisms of action and new technology given the complex nature of cancer progression [24]. Although the nanocarriers possess versatility, there remains a knowledge gap that needs to be filled before translating the preclinical findings to clinical trials [204].

In reporting recent nanotechnological advances that involves cisplatin, a recently adopted strategy for the improvement of nanocarrier drug function was the use of ferroptosis in which, the UV -catalysed Fenton reaction produced hydroxyl radicals that upregulated apoptosis in the tumour cells [205]. An iron-based compound was loaded into a mesoporous-silicon nanocarrier in which, cisplatin was attached to the functionalised silica surface. Since the UV light did not penetrate deep enough into the nanoparticles, there was up-conversion to near infrared waves and to make it more accessibility to light, and a folate-receptor targeted lipid membrane was added as an outer coating. The modified treatment attenuated triple negative breast cancer in a mouse model [205]. Another recent intervention is a novel linear dendritic polymer containing cisplatin and norcantharidin prodrug conjugates that were linked to PEG 2000 and grafted to the hydroxyl terminals in another dendritic polycarbonate core [206]. These had the ability to self-assemble into multi-micelles in solution which were effective in attenuating H22 (hepatocellular carcinoma) - bearing tumours in a mouse model with enhanced EPR effect which prolonged plasma circulation half-life [206]. They reported up to a 78% tumour inhibition rate and alleviation of myelosuppression that develops with free cisplatin treatment [206]. An innovative functionalised magnetic nanocomposite carrying nano-chitosan and cisplatin conjugates was reported to have enhanced biocompatibility and effective cervical cancerous tumour targeting when a specific magnetic field was applied [207]. Another recent addition is the manganese dioxide-structured nanoparticles loaded with Pt prodrug conjugates that are redox and tumour microenvironment-responsive [208]. These nanoparticles are able to detect cancers with an on/off switch that correlates with magnetic resonance in response to reducing agents such as glutathione (GSH) [208]. Another recent experimental nanotool is a mesoporous organosilica nanoparticles coated with a lipid membrane (known as lipid coated nanocages) to allow good biocompatibility for internalization [209]. It is achieved through exploiting electrostatic interactions that assist in the upload of hydrophobic drugs such as cisplatin allowing greater penetration across the oppositely charged organosilica external surface. These nanocages have proven success in rapid uptake with high hemocompatibility and an excellent colloidal stability [210,211]. These are lipid-shielded nanoparticles that are readily breakable for efficient drug release with the disruption of the di-sulphide bridges in the organosilica framework after internalization into the tumour cells [209]. Another new addition to the nano-sized drug carriers is an exosome loaded with ultrasmall Pt nanoparticles to achieve high drug concentrations at tumour sites to counteract unspecific drug accumulation which increases toxicity in healthy cells. This is a novel hybrid bioartificial system that self-induces the exosome uptake and rapid targeted internalization thus reducing the toxic effect and enhancing the anti-proliferative effects in the tumours [210].

When comparing the present study with those that have been published in the recent years on the same topic, there wasn't a publication that was similar to this [[212], [213], [214], [215]]. Therefore they could not be directly compared with our study. There are meta-analyses on the topic of nanotechnology in anticancer treatment with cisplatin, but we did not come across any similar studies. However, there is a large amount of research carried out in the search of a potent therapeutic formulation from the existing anticancer drug cohort. With the recent tumour-centred developments based upon the nanocarriers, the success rate of translating mouse studies to the clinic has been low. There are about 15 different nano-formulations that are undergoing clinical trials as anticancer therapeutics, only a few have obtained US FDA approval [216]. Among the cutting-edge research that is continuing at present are microfluidic human-tumour-on chips and the 3D *in vitro* models [216]. Some innovative models are based upon the EPR effect to overcome unspecific tumour targeting [217]. The EPR effect has been useful in reaching the tumour targets by producing leaky vasculature, that promotes passive drug delivery, and active targeting has been achieved with new nanoparticles camouflaged in either cancer cell membranes, stem cell membranes or normal cell membranes [218,219]. The other methods are coating the nanoparticles with antibodies, proteins, peptides, small molecules, and DNA aptamers that can be attracted to the tumours through the receptors that are overexpressed in most of the tumour cells [220]. Further, the nanoparticles are encapsulated within the cellular organelles such as mitochondria, ER, Golgi bodies, lysosomes, and exosomes [221]. There are other studies that have explored covering the nanoparticles with immune cell membranes such as RBC, WBC, and T lymphocytes [222,223]. Such decoy strategies have been successful to some extent although once the chemotherapies have reached phase II or III trials, the failure rate has been as high as 86% [216].

The nanocarrier-delivered cisplatin to various preclinical cancer models have demonstrated (i) reduced cell viability in the cancer cell lines, (ii) lowering of the IC50 values in the cancer cell lines (iii) a conspicuous reduction in the tumour volume (iv) high accumulation of Pt in the tumours, (v) low biodistribution of Pt in the vital organs, (vi) displaying high biosafety of the nanocarriers by suppressing certain haematological inflammatory biomarkers and (vii) minimising the loss of body weight that will otherwise necessitate premature euthanasia of the mice. Therefore, this study delineates the many advantages of cisplatin use in cancer therapy (Fig. 15).

This study has broad implications for cisplatin use in cancer therapy. This meta-analysis clearly indicates the significant advantages of using nanocarriers for cisplatin delivery to the tumours and provide support for clinical studies that could demonstrate the efficacy of nanocarrier-delivery as opposed to the injection of free cisplatin. There is a valid translational potential for the incorporation of nanocarrier-cisplatin systems in future cancer treatment modalities.

## Ethics approval and consent to participate

Not applicable.

## Consent for publication

All authors of this review have consented for publication.

## Funding

No funding has been received.

## Data availability statement

The data of this paper will be deposited in the Victoria University Data Repository, once published.

## CRediT authorship contribution statement

**Ranmali Ranasinghe:** Writing – original draft, Visualization, Validation, Software, Resources, Methodology, Investigation, Formal analysis, Conceptualization. **Michael Mathai:** Writing – review & editing, Supervision. **Mohammed Abdullah Alshawsh:** Writing – review & editing, Supervision. **Anthony Zulli:** Writing – review & editing, Supervision, Resources.

## Declaration of competing interest

The authors declare that they have no known competing financial interests or personal relationships that could have appeared to influence the work reported in this paper.
