# Supplementary material for: Nanocarrier-mediated cancer therapy with cisplatin: A meta-analysis with a promising new paradigm
Source: Heliyon. 2024 Mar 27;10(7):e28171. doi: 10.1016/j.heliyon.2024.e28171 (PMC11747978; doi:10.1016/j.heliyon.2024.e28171)
Supplement: Multimedia component 1 [file mmc1.docx]

SUPPLEMENTARY MATERIAL – *HELIYON-e28171*

**TABLE S1 – The characteristics of the included studies.**

| **Study #** | **Year** | **First Author** | **Type of Nanocarrier** | **Na Name of Nanocarrier** | **Combined therapy or treatment** | **Cancer type** | **Mouse model** | **Sex** | **Age (weeks)** | **Duration of study (days)** | ***In vitro* cell line** | **Ref** |
| --- | --- | --- | --- | --- | --- | --- | --- | --- | --- | --- | --- | --- |
| 1 | **2022** | **Ali Moammeri** | **Lipid based** | **Decorated niosomal nanocarriers** | **Cisplatin +Epirubicin** | **Breast cancer** | **BALB/c** | **F** | **6-8** | **20** | **SKBR3**  **4T1** | **[16]** |
| 2 | **2020** | **Barbara Bortot** | **Lipid based** | **Cisplatin-loaded biodegradable nanoparticles (Cis-NP)** | **Cisplatin** | **Epithelial ovarian cancer** | **Athymic nude-Foxn1nu mice** | **F** | **4-6** | **14** | **SKOV3 SKOV3-luc** | **[8]** |
| 3 | **2015** | **Chun-Ying Qu** | **Lipid**  **based** | **Hyaluronic acid (HA)-coated 5-FU-stearic acid prodrug and CDDP-loaded NLC (HA-FU/C-NLC)** | **Cisplatin +5-fluorouracil** | **Gastric cancer** | **BALB/c nude mice** | **NS** | **4** | **21** | **BGC823** | **[20]** |
| 4 | **2018** | **Feng Yang** | **Lipid based** | **Hyaluronic acid (HA) containing lipid nanoparticles (NPs)** | **Cisplatin + Sorafenib** | **Gastric cancer** | **BALB/c nude mice** | **NS** | **6-8** | **21** | **MKN28 SGC7901** | **[22]** |
| 5 | **2013** | **Shutao Guo** | **Lipid based** | **Lipid-coated pure cisplatin nanoparticles** | **Cisplatin** | **Melanoma** | **Athymic nude mice** | **F** | **5-6** | **14** | **A375M A375M-GFP** | **[77]** |
| 6 | **2014** | **Shutao Guo** | **Lipid based** | **1,2-dioleoyl-sn-glycero-3 -phosphate (DOPA) and lipid bilayer-coated cisplatin nanoparticles** | **Cisplatin** | **Melanoma** | **Athymic nude mice** | **F** | **5-6** | **34** | **1205Lu cells** | **[78]** |
| 7 | **2020** | **Xin Zeng** | **Lipid based** | **Platinum nanoparticles (Pt NPs)** | **Cisplatin + Dauno-rubicin** | **Chronic myelogenous leukemia and hepatocellular carcinoma** | **BALB/c nude mice** | **M/F** | **4-6** | **20** | **K562 HepG2 cell line** | **[79]** |
| 8 | **2020** | **Yonglong Jin** | **Lipid based** | **CIS-DOXp-loaded nanocarriers** | **Cisplatin + Doxorubicin** | **Lung cancer** | **BALB/c** | **F** | **NS** | **18** | **A549 or A549/CIS** | **[23]** |
| 9 | **2021** | **Qiao Jin** | **Lipid based** | **Dual threat hybrid artemisinin platinum (ArtePt) synthesized to combine chemodynamic therapy (CDT) with chemotherapy-[NP(ArtePt)]** | **Cisplatin +chemodynamic therapy** | **Hepatocellular carcinoma** | **BALB/c nude mice** | **F** | **6-8** | **10** | **7404 (liver cancer cell line) 7404DDP (cisplatin-resistant liver cancer cell line)** | **[80]** |
| 10 | **2017** | **Shuting Xu** | **Lipid based** | **Cisplatin with vorinostat (a FDA-approved histone deacetylase inhibitor) to form a supramolecular conjugate, which can further self-assemble into nanoparticles** | **CDDP +Vorinostat (SAHA)** | **Non-Small Cell Lung Cancer (NSCLC)** | **BALB/c nude mice** | **F** | **5** | **33** | **A549 A549/DR** | **[81]** |
| 11 | **2017** | **Shuyi Li** | **Lipid based** | **Nanoscale micelles encapsulating ethacraplatin, a conjugate of cisplatin and ethacrynic acid (an effective GSTs inhibitor),** | **Cisplatin + Ethacraplatin** | **Cisplatin-resistant liver cancer** | **NOD SCID mice** | **F** | **NS** | **30** | **BEL7404**  **BEL 7404-CP20** | **[82]** |
| 12 | **2017** | **Bo Liu** | **Polymeric** | **Trans-activating transcriptional activator (TAT)-modified polyethylene glycol-distearoyl-phosphatidyl ethanolamine solid lipid nanoparticles (SLNs) or [TAT PTX/TOS-CDDP SLNs)** | **Paclitaxel (PTX) and α-tocopherol succinate-cisplatin prodrug** | **Cervical cancer** | **BALB/c nude mice** | **F** | **4-6** | **21** | **HeLa cells** | **[25]** |
| 13 | **2020** | **Cho Rong Park** | **Polymeric** | **CDDP–Human Serum Albumin complex (HSA–CDDP) nanocarrier mediated by the secreted protein acidic and rich in cysteine (SPARC)** | **Cisplatin bound to HSA** | **Glioblastoma** | **BALB/c nude mice** | **M** | **5** | **14** | **U87MG U87MG-shSPAR** | **[83]** |
| 14 | **2021** | **Haijun Wang** | **Polymeric** | **Human epidermal growth factor receptor 2 (Her2)-specific dimeric affibody (ZHer2) mesoporous Polydopamine/MnO2/polydopa--mine nanoparticles** | **Cisplatin + Radiotherapy** | **Ovarian cancer** | **BALB/c nude mice** | **F** | **4-6** | **14** | **SKOV-3** | **[84]** |
| 15 | **2021** | **Jiaxi Xu** | **Polymeric** | **A sequentially responsive Pt (IV) nanogels are fabricated by copolymerization.** | **Cisplatin** | **A549/DDP tumour** | **BALB/c nude mice** | **F** | **5-6** | **11** | **A549 A549/DDP** | **[26]** |
| 16 | **2017** | **Jing Tian** | **Polymeric** | **CP prodrug (CPP) formulated poly (lactic-co-glycolic acid)-poly (ethylene glycol) (PLGA-PEG) nanoparticles (NPs)** | **Cisplatin + Docetaxel** | **Lung cancer** | **Athymic nude mice** | **F** | **6-8** | **15** | **H460 344SQ cell line** | **[85]** |
| 17 | **2013** | **Jinrong Peng** | **Polymeric** | **pH-thermal dual responsive nanogel** | **Cisplatin +Doxorubicin** | **Breast cancer** | **BALB/c** | **F** | **4-6** | **NS** | **4T1**  **HeLa cells** | **[86]** |
| 18 | **2015** | **Joseph Della Rocca** | **Polymeric** | **Nanoparticles (NP) comprised of polysilsesquioxane (PSQ) polymer crosslinked by a cisplatin prodrug (Cisplatin-PSQ)** | **Cisplatin and Radiotherapy** | **Non-small cell lung cancer** | **nu/nu** | **M** | **4-6** | **30** | **NCI-H460 and A549** | **[87]** |
| 19 | **2020** | **Le He** | **Polymeric** | **pH/redox dual-sensitive polymer hybrid micelles** | **Platinum (IV)-coordinate polymeric prodrugs and D-α-tocopheryl polyethylene glycol succinate (TPGS)** | **Multidrug resistance (MDR) / primary cause of failure in oncotherapy** | **H22**  **tumour-bearing mice** | **M** | **NS** | **NS** | **A549**  **A549/DDP** | **[88]** |
| 20 | **2020** | **Min Sun** | **Polymeric** | **Multifunctional nanogel (designated as Valproate-D-Nanogel) from copolymerization of carboxymethyl chitosan and diallyl disulfide** | **Cisplatin** | **Lung adeno carcinoma** | **BALB/c nude mice** | **NS** | **NS** | **12** | **A549 /DDP** | **[27]** |
| 21 | **2013** | **Stephanie M. Cohen** | **Polymeric** | **(Hyaluronan [HA])–conjugated cisplatin (HA-cisplatin)** | **Cisplatin** | **Head and Neck squamous cell carcinoma (HNSCC)** | **nu/nu mice** | **F** | **4-6** | **42** | **MDA-1986 HNSCC cells** | **[89]** |
| 22 | **2016** | **Yunkun Li** | **Polymeric** | **pH/redox dual-responsive theranostic supramolecular PEGylated dendritic systems (TSPDSs).** | **Cisplatin + Doxorubicin** | **Lung cancer** | **BALB/c nude mice** | **M** | **NS** | **21** | **A549** | **[90]** |
| 23 | **2017** | **Urvashi Aggarwal** | **Polymeric** | **Cisplatin loaded poly-caprolactone/ chitosan composite electrospun nanofiber** | **Cisplatin** | **Cervical cancer** | **Swiss albino mice** | **F** | **NS** | **21** | **Erlich ascites carcinoma (EAC) cell lines** | **[91]** |
| 24 | **2017** | **Peng Zhang** | **Polymeric** | **Admantyl (Ad)- terminated (ß poly (aspartic acid) (Ad-P(Asp)) and the ß-cyclodextrin (ß-CD)-terminated poly(2-methyl-2-oxazoline) cisplatin-rich supramolecular nanoparticles** | **Cisplatin** | **H22 tumours** | **ICR mice** | **M** | **6-7** |  | **H22** | **[92]** |
| 25 | **2020** | **Yi-Fang Zhong** | **Polymeric** | **Cy5 DNA nanostructures** | **Cisplatin** | **Lung cancer** | **Athymic nude mice** | **NS** | **NS** | **14** | **A549**  **A549cisR**  **HeLa**  **MGC-803**  **Helf** | **[93]** |
| 26 | **2020** | **Tong Yang** | **Polymeric** | **Hyaluronic acid-cisplatin/polystyrene-polymetformin (HA-CDDP/PMet) dual-prodrug co-assembled nanoparticles. [HA-CDDP/PMet NPs]** | **Cisplatin**  **Metformin** | **Lung cancer** | **C57/BL6**  **ICR** | **F** | **4-6** | **22** | **4T1**  **LLC**  **HepG2** | **[94]** |
| 27 | **2019** | **Xiaoyin Qiao** | **Polymeric** | **Controlled-release poly-lactic-co-glycolic acid (PLGA) scaffolds were fabricated by E-jet 3D printing to deliver doxorubicin (DOX) and cisplatin (CDDP) simultaneously** | **Cisplatin**  **Doxorubicin** | **Breast cancer** | **BALB/c nude mice** | **F** | **6** | **30** | **MDA-MB-231**  **NIH-3 T3** | **[95]** |
| 28 | **2018** | **Sheng Tan** | **Polymeric** | **FA and Tf modified CDDP loaded NPs (FA/Tf-CDDP-NPs)** | **Cisplatin** | **Non-small-cell lung cancer** | **C57/BL6 nude mice** | **M** | **6** | **18** | **A549**  **NC1-H460**  **HUVEC** | **[31]** |
| 29 | **2019** | **Yuxin Wang** | **Polymeric** | **Human serum albumin indocyanine green-cisplatin nanoparticles (HSA-ICG-DDP NPs)** | **Cisplatin** | **Oral squamous cell cancer (OSCC)** | **BALB/c athymic nude mice** | **NS** | **NS** | **15** | **CAF**  **HSC**  **NF**  **NCM-460** | **[96]** |
| 30 | **2021** | **Xun Liu** | **Polymeric** | **Histone deacetylase/cathepsin L-responsive acetylated azidomannose (DCL-AAM) dibenzocyclo octyne-cisplatin (DBCO-Pt) prodrug loaded nanocarrier** | **Cisplatin** | **Ovarian cancer** | **BALB/c and BALB/c nude mice** | **F** | **6-8** | **60** | **SKOV3, A549, HeLa, MCF-7, MCF-/ADR, MDA-MB-231, HepG2** | **[97]** |
| 31 | **2021** | **Kun Chen** | **Polymeric** | **Prodrug delivery system based on branched ß-(1 .3)-D-glucan. Polysaccharide nanotube modified with cisplatin embedded in the hollow cavity (BFCP).** | **Cisplatin** | **Breast cancer** | **Athymic nude mice** | **NS** | **NS** | **25** | **HeLa**  **MCF-7** | **[98]** |
| 32 | **2022** | **Guodong Ren** | **Polymeric** | **Green fluorescence carbon dots (GCDs) conjugated with epidermal growth factor (EGF) and Chlorin e6 (Ce6) in the presence of laser irradiation. [GCDs-Ce6/Pt-EGF]** | **Cisplatin and epidermal growth factor (EGF) and Chlorin e6 (Ce6)** | **Oesophageal cancer** | **BALB/c nude mice** | **NS** | **6** | **21** | **KYSE-150** | **[99]** |
| 33 | **2022** | **Fangman Cheng** | **Inorganic** | **Diselenide-bridged mesoporous organosilica nanoparticles (MON)coated with biomimetic cancer cell membrane** | **Cisplatin** | **Breast cancer** | **BALB/c** | **NS** | **4-5** | **21** | **4T1** | **[100]** |
| 34 | **2014** | **Lei Miao** | **Inorganic** | **Dioleoyl phosphatidic acid (DOPA)-gemcitabine monophosphate and DOPA coated cisplatin-precipitate nanoparticles** | **Cisplatin and Gemcitabine** | **Aggressive stroma-rich bladder cancer** | **Athymic nude mice** | **F** | **6-8** | **18** | **UMUC3 NIH 3T3** | **[101]** |
| 35 | **2019** | **Lu Zhang** | **Inorganic** | **αvβ3 integrin-targeted, cisplatin-loaded and radioisotope iodine-125 labelled spherical and rod-shaped gold nano theranostic probes (RGD-125IPt-AuNPs and RGD-125IPt-AuNRs)** | **Cisplatin + Radiotherapy** | **Non-small cell lung carcinoma** | **BALB/c nude mice** | **NS** | **NS** | **21** | **H1299** | **[102]** |
| 36 | **2021** | **Zhi-hang Zhou** | **Inorganic** | **Hollow mesoporous MnO2nanoshells/ [H-MnO2-PEG/TP nanoshells]** | **Cisplatin + Docetaxel** | **High metastatic oral squamous cell carcinoma** | **BALB/c nude mice (nu/nu)** | **NS** | **4** | **28** | **CAL 27 SCC7** | **[103]** |
| 37 | **2017** | **Adem Guven** | **Inorganic** | **Cisplatin (CDDP) encapsulated within ultra-short single-walled carbon nanotube capsules (CDDP@US-tubes)** | **Cisplatin** | **Breast cancer** | **SCID/ Beige mice** | **NS** | **NS** | **28** | **MCF-7 BCM-4272 MDA-MB-231** | **[104]** |
| 38 | **2015** | **Natividad Gomez- Roman** | **Inorganic** | **Cucurbit [7] uril-encapsulated cisplatin (cisplatin@CB [7])** | **Cisplatin** | **Ovarian cancer** | **Athymic nude mice -MF1 (nu/nu)** | **F** | **6-8** | **13** | **A2780 cell line** | **[105]** |
| 39 | **2020** | **Ge Chen** | **Lipid based +polymeric** | **Nitroimidazoles (NI) and Hyaluronic acid (HA) co-decorated, cisplatin (DDP) loaded polymeric nanoparticles (PNPs) (NI/HA-DDP-PNPs) and lipidpolymer hybrid nanoparticles (LPNs) (NI/HA-DDP-LPNs)** | **Cisplatin** | **Lung cancer** | **BALB/c nude mice** | **F** | **6-8** | **21** | **A549 A549/DDP** | **[106]** |
| 40 | **2022** | **Guannan Zhou** | **Lipid based +polymeric** | **Milk-derived exosome** | **Cisplatin** | **Ovarian carcinoma** | **Athymic nude (nu/nu)** | **F** | **5-6** | **21** | **A2780CP** | **[107]** |
| 41 | **2017** | **Jiying Yang** | **Lipid based +polymeric** | **Folate-polyethylene glycol PEG-DSPE and drugs-loaded nanostructured lipid carriers (NLCs)- [FA-DDP/PTX NLCs]** | **Cisplatin + Paclitaxel** | **Head and Neck cancer** | **BALB/c** | **NS** | **6-8** | **21** | **Fa Du cells** | **[108]** |
| 42 | **2013** | **Natalia V Nukolova** | **Lipid based +polymeric** | **Soft block copolymer nanogels with ionic cores serving as a reservoir for cisplatin (loading 35%) and a synthetic analogue of LHRH conjugated to the nanogels via poly (ethylene glycol) spacer** | **Cisplatin** | **Ovarian cancer** | **Athymic nu/nu mice** | **F** | **4** | **23** | **A2780 SKOV-3** | **[109]** |
| 43 | **2020** | **Tao Yu** | **Lipid based +polymeric** | **Hyaluronic acid (HA)-based nano-carrier - a CD44-targeting anti-cancer drug delivery system- [HA-DOX-CDDP micelles]** | **Cisplatin + Doxorubicin** | **Breast cancer** | **BALB/c** | **F** | **4-5** | **30** | **4T1 (CD44+) and NIH-3T3 (CD44-)** | **[110]** |
| 44 | **2016** | **Yang Xiong** | **Lipid based +polymeric** | **Polyglutamic acid (PGA) complexed with cationic polymeric metformin (polymet) and stabilized with cationic liposomes composed of DOTAP (2,3-Dioleoyloxy-propyl)-trimethylammonium/ Cholesterol/DSPE-PEG-anisamide aminoethyl nanoparticles** | **Cisplatin + Metformin** | **Non-small cell lung cancer** | **Athymic nude mice** | **F** | **NS** | **12** | **H460** | **[111]** |
| 45 | **2020** | **Yuan Hong** | **Lipid based +polymeric** | **CDDP prodrug and CUR co-encapsulated layer-by-layer nanoparticles (CDDP-PLGA/CUR LBL NPs)** | **Cisplatin + Curcumin** | **Non-small cell lung cancer** | **BALB/c nude mice** | **NS** | **NS** | **21** | **A549, NCI-H1299 or BEAS-2B** | **[112]** |
| 46 | **2012** | **Haiqin Song** | **Lipid based +polymeric** | **A polymeric cisplatin (IV) prodrug cross-linked micelles (M(Pt (IV))** | **Cisplatin** | **Murine cervical cancer (U14 cells)** | **Kunming mice** | **NS** | **6** | **14** | **HeLa**  **MCF-7**  **SKOV-3** | **[113]** |
| 47 | **2015** | **Zhoufeng Wang** | **Lipid based +polymeric** | **Reduction-sensitive therapeutic micelles consisting of polyethylene glycol-poly-(l-glutamic acid) (PEG-PLG), and conjugate with dithiodipropionic-Pt (IV) (DTDP-Pt).** | **Cisplatin** | **Human ovarian carcinoma** | **BALB/c nude mice** | **F** | **4-6** | **18** | **SKOV-3** | **[114]** |
| 48 | **2016** | **Guilian Zhang** | **Lipid based +polymeric** | **Folic Acid (FA)- containing polyethylene glycol (PEG)-distearoylphosphatidyl ethanolamine (DSPE) (FA-PEG-DSPE) modified, CIS-loaded non-structured lipid carriers (NLCs). [(FA-CIS-NLCs)]** | **Cisplatin** | **Cervical cancer** | **BALB/c nude mice** | **NS** | **6-8** | **15** | **HeLa cells** | **[115]** |
| 49 | **2015** | **Ming-qiang Li** | **Lipid based +polymeric** | **Cisplatin-loaded LHRH-modified dextran nanoparticles (Dex-SA-CDDP-LHRH)** | **Cisplatin** | **Breast cancer** | **BALB/c**  **Kunming mice** | **F**  **M** | **5-6** | **20** | **4T1** | **[116]** |
| 50 | **2015** | **Dongfang Zhou** | **Lipid based +polymeric** | **Canthaplatin, a Pt (IV) pro-drug of cisplatin and a protein phosphatase 2A (PP2A) inhibitor (4-(3-carboxy-7-oxa- bicyclo [2.2.1] heptane-2-carbonyl) piperazine-1-carboxylic acid tert-butyl ester), was designed and delivered using PEG-b-PLGA micelles** | **Cisplatin** | **Lung cancer** | **Kunming (KM) mice and nude BALB/c mice** | **NS** | **6-8 and 4-6** | **32** | **A549 or A549/DDP** | **[117]** |
| 51 | **2020** | **Shu-Jyuan Yan** | **Inorganic+**  **polymeric** | **Superparamagnetic iron oxide (SPIO) and poly (sodium styrene sulfonate) (PSS) at the core and a layer-by-layer shell with cisplatin (CDDP), with methotrexate – human serum albumin conjugate (MTX−HSA conjugate) [SPIO@PSS/CDDP/HSA−MTX nanoparticles (NPs)].** | **Cisplatin and methotrexate** | **Lung cancer** | **BALB/c AnN. Cg- Foxnlnu/Crl Narl-nude mice** | **F** | **4** | **24** | **NCI-H661 A549** | **[118]** |
| 52 | **2015** | **Ti Zhang** | **Inorganic +polymeric** | **Hyaluronic acid (HA) and a conjugated cisplatin anticancer drug tracked by lanthanum (III) [La(III)] affinity tagging of the nanocarrier [HA-Pt-La nanoparticles]** | **Cisplatin** | **HNSCC** | **nu/nu** | **F** | **NS** | **NS** | **HNSCC MDA-1986 cells** | **[119]** |
| 53 | **2020** | **Xibo Pei** | **Inorganic +polymeric** | **Dual-drug-loaded polyethylene glycol (PEG)ylated nano-graphene oxide (pGO)** | **Cisplatin + Doxorubicin** | **Squamous cell carcinoma and Breast cancer** | **Athymic nude mice** | **F** | **6** | **21** | **CAL-27 MCF-7** | **[120]** |
| 54 | **2016** | **Efstathia Voulgari** | **Inorganic+ polymeric** | **Poly (methacrylic acid)-g-poly (ethyleneglycol methacrylate) polymers as in situ coating agents for magnetite nanocrystallites.** | **Cisplatin +magnetic field** | **Human colon adenocarcinoma** | **CB17 SCID mice** | **F** | **6** | **38** | **HT-29** | **[121]** |
| 55 | **2022** | **Meng Si** | **Polymeric** | **Poly (lactide-co- glycolide)-poly (ethylene glycol)- triblock copolymer hydrogel containing doxorubicin and cisplatin** | **CDDP+DOX** | **Osteosarcoma** | **BALB/c nu/nu nude mice** | **M** | **5** | **14** | ***SAOS***  ***MG63*** | **[122]** |
| 56 | **2022** | **Qianwen Li** | **Lipid-based+Polymeric** | **Estrone-targeted PEGylated Liposomal DDP (ES-SSL-DDP)** | **CDDP** | **Cervical cancer** | **BALB/C nude mice + ICR** | **F** | **4-5/6-8** | **27** | ***HeLa*** | **[32]** |
| 57 | **2021** | **Lisa Agnello** | **Polymeric** | **Polymeric nanoparticles (PNPs) that were conjugated on their surface with the epidermal growth factor receptor (EGFR) with Cl4 aptamer** | **CDDP +BODIPY505-515** | **Triple negative breast cancer** | **Athymic Nude-*Foxn1nu* mice** | **F** | **6** | **23** | ***MDA-MB-231, BT-549 or MDA-MB-231 EGFR-KO cells*** | **[123]** |
| 58 | **2021** | **Yan Chen** | **Inorganic +Polymeric** | **Chitosan oligosaccharide (COS)-coated and sialic acid (SA) receptor-targeted nano-micelles to co-deliver cisplatin (CDDP) and nitric oxide (NO) (denoted as CTP/ CDDP)** | **CDDP +NO** | **Lung cancer** | **BALB/c nude mice** | **F** | **6-8** | **24** | ***4T1,***  ***MCF-7*** | **[124]** |
| 59 | **2019** | **M Satpathy** | **Inorganic + polymeric** | **An amphiphilic polymer coated magnetic iron oxide nanoparticle was conjugated with near infrared dye labelled HER2 affibody and chemotherapy drug cisplatin** | **CDDP** | **Ovarian cancer** | **Athymic nude mice** | **F** | **6-8** | **21** | ***SKOV-3*** | **[125]** |
| 60 | **2018** | **Xiaoxu Zhao** | **Inorganic** | **Gold nanoparticles (AuNPs)** | **CDDP** | **Colorectal cancer** | **BALB/c nude mice** | **F** | **6-8** | **14** | ***CAF***  ***SW620*** | **[126]** |
| 61 | **2019** | **Yingli Zhang** | **Lipid-based + Polymeric** | **Lipid–polymer hybrid nanoparticles (DDPLPN)** | **CDDP** | **Ovarian cancer** | **BALB/c nude mice** | **F** | **NS** | **18** | ***SKOV3*** | **[33]** |
| 62 | **2020** | **Xiaojuan Zhang** | **Inorganic** | **Microporous organosilica shell-coated cisplatin nanoplatform** | **CDDP +Acriflavine** | **Lung cancer** | **BALB/c and BALB/c nude mice** | **F** | **4-6** | **24** | ***A549***  ***4T1***  ***CT26*** | **[29]** |
| 63 | **2021** | **Guiying Jiang** | **Inorganic +polymeric** | **TMTP1-modified, cisplatin and paclitaxel prodrugs co-loaded nanodrug** | **CDDP +Paclitaxel** | **Cervical cancer** | **BALB/c nude mice** | **F** | **6** | **35** | ***SiHa***  ***HeLa*** | **[127]** |
| 64 | **2016** | **Michael W Sim** | **Polymeric** | **HA -Cisplatin** | **CDDP** | **Head and neck squamous cell cancer** | **Athymic nude mice** | **F** | **4-6** | **63** | ***Laryngeal cancer cells*** | **[128]** |
| 65 | **2021** | **Zhiyuan Zhang** | **Inorganic +polymeric** | **A nanocomposite using graphene Oxide and bonded to magnetic nanocomposites (Fe3O4@rGO-G-PSEA) with Poly (hydroxyethyl methacrylate grafted to succinic anhydride).** | **CDDP + Metformin** | **Hepatocellular carcinoma** | **BALB/c nude mice** | **NS** | **4-5** | **22** | ***HepG2***  ***Caco-2*** | **[129]** |
| 66 | **2018** | **Chia-En Chang** | **Lipid-based** | **A pluronic lecithin organogel (PLO)** | **CDDP + Docetaxel** | **Ovarian cancer** | **BALB/c and BALB/c nude mice** | **F** | **5** | **42** | ***SKOV3*** | **[130]** |
| 67 | **2020** | **Bingtao Zhai** | **Lipid-based + Polymeric** | **An amino-terminal fragment (ATF) peptide-targeted liposome carrying β-elemene (ATF24-PEG-Lipo-β-E)** | **CDDP** | **Bladder cancer** | **Nude mice** | **F** | **6-8** | **16** | ***KU-19-19*** | **[131]** |
| 68 | **2018** | **Li Zhang** | **Lipid-based + Polymeric** | **PGA–Asp–maleimide–cisplatin–peptide complex (PAMCP), loaded with CDDP and conjugated with the transferrin receptor (TFR)-targeting peptide through a maleimide functional linker** | **CDDP** | **Cervical cancer** | **BALB/c nude mice and Kun Ming (KM) mice** | **F** | **6 and 5-7** | **14** | ***HeLa***  ***HUVEC*** | **[132]** |
| 69 | **2013** | **Shanthi Ganesh** | **Polymeric** | **Near infrared (NIR) dye-loaded and cisplatin loaded in CD44 targeted hyaluronic acid (HA) nanoparticles (NPs)** | **CDDP + siRNA** | **NSCLC** | **Nude mice** | **NS** | **5-6** | **Not specified** | ***A549***  ***H69AR*** | **[133]** |
| 70 | **2020** | **Yanqing Wang** | **Polymeric** | **CDDP-poly (lactic-co-glycolic acid) (CDDP-PLGA) polymer compound stent** | **CDDP** | **Ovarian cancer** | **BALB/c nude mice** | **F** | **4-6** | **49** | ***SKOV3***  ***A2780*** | **[134]** |
| 71 | **2020** | **Qingxia Fu** | **Lipid-based polymeric** | **CIS and 5-FU co-encapsulated lipid–- polymer hybrid nanoparticles decorated with TAB (TAB-CIS/5-FU LPHNs)** | **CDDP + fluoropyrimidine (5-FU)** | **Oesophageal cancer** | **BALB/c-nude mice** | **NS** | **4** | **21** | ***BE3*** | **[135]** |
| 72 | **2018** | **Ying Xie** | **Lipid-based polymeric** | **PCX/anti-miR-210 nanoparticles** | **CDDP + Paclitaxel** | **Cholangiocarcinoma** | **Athymic nu/nu mice** | **M** | **6** | **22** | ***Mz-ChA-1 cells*** | **[136]** |
| 73 | **2018** | **Zheng Wei** | **Inorganic + polymeric** | **Pt-loaded, polyethylene glycol-modified graphene quantum dots** | **CDDP** | **Oral squamous cell carcinoma** | **BALB/cJNJu-Foxn1nu/Nju mice** | **M** | **4** | **15** | ***HSC3, SCC4, CAL-27*** | **[137]** |
| 74 | **2020** | **Basavaraj R Patil** | **Inorganic + polymeric** | **A cisplatin carrier polymer prepared by grafting equimolar polyethylene glycol (PEG550) and aminoethanol to the poly(dichlorophosphazene) backbone. (Polycisplatin)** | **CDDP** | **Human alveolar basal epithelial carcinoma and human gastric cancer** | **BALB/C nude mice and ICR mice** | **M** | **5** | **35** | ***MKN-28***  ***A549*** | **[138]** |
| 75 | **2020** | **Zhen Liang** | **Inorganic** | **A multi-layered nano-platform containing cisplatin prodrug (CISP), vinorelbine (VNR) and retinoic acid (ATRA) named CISP/VNR/ATRA MLNP** | **CDDP + vinorelbine + retinoic acid** | **NSCLC** | **BALB/c nude mice** | **NS** | **6-8** | **18** | ***A549/CIS***  ***BEAS-2B*** | **[28]** |
| 76 | **2016** | **Haijun Yu** | **Lipid-based + Polymeric** | **Triple-layered pH-responsive micelleplex loading siRNA and alkylated cisplatin prodrug self-assembled from poly(ethylene glycol)-block-poly(aminolated glycidyl methacrylate)-block-poly(2-(diisopropyl amino) ethyl methacrylate) (PEG-b-PAGA-b-PDPA) triblock copolymers** | **CDDP + siRNA+p65** | **Breast cancer** | **Nude mice** | **F** | **6** | **24** | ***4TI***  ***A549*** | **[139]** |
| 77 | **2016** | **Xiaojie Lv** | **Inorganic** | **Covalently conjugated 6-mercaptopurine (6MP) onto the surface of mercapto-modified mesoporous silica nanoparticles (MSNS) to form MSNS-6MP and loaded CDDP into the holes on the surface of MSNS-6MP to form MSNS-6MP/CDDP** | **CDDP** | **Murine sarcoma** | **ICR** | **M** | **6** | **13** | ***S180*** | **[140]** |
| 78 | **2021** | **Baohua Wang** | **Inorganic** | **Cisplatin pro-drug and paclitaxel co-loaded nanoparticles: DDP-P/PTX NPs** | **CDDP + Paclitaxel** | **Lung cancer** | **BALB/c nude mice** | **F** | **4-6** | **21** | ***A549***  ***A549/DDP*** | **[37]** |
| 79 | **2021** | **Maofan Zhang** | **Lipid-based polymer** | **Hydrophobic platinum prodrug, co-delivered with etoposide in a nanoparticle** | **CDDP +Etoposide** | **Lung cancer** | **Nude mice** | **M** | **6-8** | **20** | ***H460***  ***344SQ*** | **[141]** |
| 80 | **2016** | **Fangyuan Zhou** | **Inorganic** | **Fluorescence gold nanoclusters (GNC) conjugated with a cisplatin prodrug and folic acid (FA) (FA-GNC-Pt)** | **CDDP** | **Breast cancer** | **BALB/c nude mice** | **F** | **4-6** | **22** | ***4T1*** | **[142]** |
| 81 | **2014** | **Shutao Guo II** | **Lipid-based +Inorganic** | **Lipid-coated iodinated CDDP nanoparticles (LPI -NPs)** | **CDDP** | **Melanoma** | **Athymic nude mice** | **F** | **5-6** | **45** | ***A375***  ***1205Lu*** | **[143]** |
| 82 | **2018** | **Maofan Zhang** | **Polymeric** | **Poly(lactic-co-glycolic acid)-poly(ethylene glycol) (PLGA-PEG) NPs** | **CDDP + Wortmannin** | **Ovarian cancer** | **Nude mice** | **F** | **6-8** | **16** | ***A2780***  ***A2780CIS*** | **[144]** |
| 83 | **2018** | **Jing Tian** | **Polymeric** | **Co-encapsulation of cisplatin (CP) and PTX in PLGA-PEG NPs.** | **CDDP+**  **Paclitaxel** | **NSCLC** | **Athymic nude mice** | **F** | **NS** | **25** | ***344SQ***  ***H460***  ***H69AR***  ***A549*** | **[18]** |
| 84 | **2018** | **Courtney Penn** | **Polymeric** | **G5-dendrimer nanoparticles using methotrexate G5-methotrexate nanoparticles (G5-MTX-NPs)** | **CDDP** | **Ovarian/**  **peritoneal**  **cancer** | **C57BL/6** | **NS** | **NS** | **NS** | ***ID8*** | **[145]** |
| 85 | **2018** | **Xiaomeng**  **Wan** | **Polymeric** | **Polymeric micelle system based on amphiphilic block copolymer poly(2-oxazoline)s (POx) poly(2-methyl-2-oxazoline-block-2-butyl-2-oxazoline-block-2-methyl-2-oxazoline) (P(MeOx-bBuOx-b-MeOx) with alkylated cisplatin prodrug** | **CDDP** | **Lung cancer** | **Athymic nude mice** | **F** | **6-8** | **90** | ***H69AR***  ***A549*** | **[146]** |
| 86 | **2018** | **Meng Zhang** | **Polymeric** | **HK2 shRNA was encapsulated in a polyethylene glycol-polyethylenimine copolymer modified with the FSH β 33–53 or retro-inverso FSH β 33–53 peptide.** | **CDDP** | **Ovarian cancer** | **Nude mice** | **F** | **NS** | **13** | ***A2780*** | **[147]** |
| 87 | **2018** | **Shichao Ai** | **Polymeric** | **An integrin targeted drug delivery system iRGD-heparin nanocarrier (iHP)** | **CDDP** | **Gastric cancer** | **SCID nu/nu mice** | **M** | **5** | **24** | ***MKN-45P*** | **[148]** |
| 88 | **2016** | **Yongwei Hao** | **Inorganic** | **Redox- and pH-responsive manganese dioxide nanosheets functionalized by hyaluronic acid [MnO2 /HA/CDDP]** | **CDDP** | **Lung cancer** | **NS** | **NS** | **NS** | **12** | ***A549*** | **[149]** |
| 89 | **2018** | **Yao Cheng** | **Lipid based** | **CDDP and CUR co-loaded liposomes (CDDP/CUR-Lip)** | **CDDP Curcumin** | **Hepatocellular carcinoma** | **BALB/c nude mice + Kunming mice** | **F** | **6-8** | **28** | **HepG2, H22** | **[39]** |
| 90 | **2018** | **Guowen Wang** | **Lipid based** | **RGD-modified PTX and cisplatin (CDDP) loaded LPNs (RGD-ss-PTX/CDDP LPNs)** | **CDDP, Paclitaxel** | **Lung cancer** | **Balb/c-nude mice** | **F** | **8-10** | **21** | ***A549****,* **NCI-H1299** | **[38]** |
| 91 | **2017** | **Ki Hyun Bae** | **Polymeric** | **Hyaluronic acid-EGCG conjugate micellar nanocomplex** | **CDDP, Epigallocatechin-3-O-gallate (EGCG)** | **Ovarian cancer** | **SCID-beige mice** | **F** | **6-8** | **35** | ***SKOV-3, HCT116, HEK293T*** | **[150]** |
| 92 | **2023** | **María Sancho-Albero** | **Inorganic** | **Breakable cage-like organosillica particles (ssOSCs)** | **Pt(dach)Cl2(OH)2** | **Malignant pleural mesothelioma** | **Athymic nude mice** | **F** | **7** | **35** | ***HeLa*** | **[151]** |
| 93 | **2023** | **Francisco Silva** | **Inorganic polymeric** | **Pt containing AuNP-BBN-Pt (Pt = Pt1-Pt3) nanoparticles** | **Pt (iv) prodrug** | **Prostate cancer** | **Balb/c-Nude mice** | **NS** | **10-12** | **40** | ***PC3*** | **[152]** |
| 94 | **2023** | **Mingli Wei** | **Polymeric** | **Linear−dendritic polymer, G1(PPDC) constructed**  **into raspberry-like multicell clusters in solution** | **CDDP) and Norcantharidin (NCTD** | **Hepato**  **cellular carcinoma (HCC)** | **Kunming**  **mice** |  | **NS** | **24** | ***HepG2,***  ***A549,***  ***NIH3T3, H22*** | **[153]** |
